# *Babesia gibsoni* Whole-Genome Sequencing, Assembling, Annotation, and Comparative Analysis

**DOI:** 10.1128/spectrum.00721-23

**Published:** 2023-07-11

**Authors:** Qin Liu, Xing-Ai Guan, Dong-Fang Li, Ya-Xin Zheng, Sen Wang, Xue-Nan Xuan, Jun-Long Zhao, Lan He

**Affiliations:** a State Key Laboratory of Agricultural Microbiology, College of Veterinary Medicine, Huazhong Agricultural University, Wuhan, Hubei, China; b Key Laboratory of Preventive Veterinary Medicine in Hubei Province, Wuhan, Hubei, China; c National Research Center for Protozoan Diseases, Obihiro University of Agriculture and Veterinary Medicine, Obihiro Hokkaido, Japan; Hubei University of Medicine

**Keywords:** apicomplexan, *Babesia gibsoni*, protozoan, genome sequencing, genome assembly, comparison analysis, apicomplexan parasites

## Abstract

The intracellular protozoan parasite Babesia gibsoni infects canine erythrocytes and causes babesiosis. The hazards to animal health have increased due to the rise of B. gibsoni infections and medication resistance. However, the lack of high-quality full-genome sequencing sets has expanded the obstacles to the development of pathogeneses, drugs, and vaccines. In this study, the whole genome of *B. gibsoni* was sequenced, assembled, and annotated. The genomic size of *B. gibsoni* was 7.94 Mbp in total. Four chromosomes with the size of 0.69 Mb, 2.10 Mb, 2.77 Mb, and 2.38 Mb, respectively, 1 apicoplast (28.4 Kb), and 1 mitochondrion (5.9 Kb) were confirmed. KEGG analysis revealed 2,641 putative proteins enriched on 316 pathways, and GO analysis showed 7,571 annotations of the nuclear genome in total. Synteny analysis showed a high correlation between *B. gibsoni* and B. bovis. A new divergent point of *B. gibsoni* occurred around 297.7 million years ago, which was earlier than that of *B. bovis*, B. ovata, and B. bigemina. Orthology analysis revealed 22 and 32 unique genes compared to several *Babesia* spp. and apicomplexan species. The metabolic pathways of *B.gibsoni* were characterized, pointing to a minimal size of the genome. A species-specific secretory protein SA1 and 19 homologous genes were identified. Selected specific proteins, including apetala 2 (AP2) factor, invasion-related proteins BgAMA-1 and BgRON2, and rhoptry function proteins BgWH_04g00700 were predicted, visualized, and modeled. Overall, whole-genome sequencing provided molecular-level support for the diagnosis, prevention, clinical treatment, and further research of *B. gibsoni*.

**IMPORTANCE** The whole genome of *B. gibsoni* was first sequenced, annotated, and disclosed. The key part of genome composition, four chromosomes, was comparatively analyzed for the first time. A full-scale phylogeny evolution analysis based on the whole-genome-wide data of *B. gibsoni* was performed, and a new divergent point on the evolutionary path was revealed. In previous reports, molecular studies were often limited by incomplete genomic data, especially in key areas like life cycle regulation, metabolism, and host-pathogen interaction. With the whole-genome sequencing of *B. gibsoni*, we provide useful genetic data to encourage the exploration of new terrain and make it feasible to resolve the theoretical and practical problems of babesiosis.

## INTRODUCTION

Babesia gibsoni, an apicomplexan member, is an obligate intracellular parasite that infects dogs and causes babesiosis. This infection can result in various clinical features including fever, hemolytic anemia, and icterus in acute infection, as well as long-lasting chronic illness (1, 2). Furthermore, B. gibsoni infection can be fatal for dogs with low immunity and underlying diseases (3), leading to considerable morbidity and mortality. As the apicomplexan family has developed an adaptive ability to survive and invade animal host cells, it has become a worldwide animal husbandry industry and public health problem (4, 5). Thus, there is an urgent need for continuous and targeted treatments or immune protection through vaccination to prevent further infection.

Detecting *B. gibsoni* by intraerythrocyte morphology in Giemsa-stained blood smears and serological tests is the most accessible strategy during clinical diagnoses. However, incorrect diagnoses often occur due to low-level and intermittent parasitemia during the chronic stage (6, 7). Additionally, cross-reaction between *B. gibsoni* and other related endoparasites leads to poor specificity of serological diagnoses (8). For molecular identification, a nested-PCR test of internal transcribed spacer 1 (ITS1) region is a regularly executed method, which was first applied based on partial 18S rRNA genes in GenBank (9–11). Although PCR detection has been improved by several specific targets (for example, the diagnostic efficiency of mitochondrial cytochrome oxidase subunit III (*cox3*) was higher than BgITS-1), the analytical sensitivity of PCR without a complete gene sequence is still troubled by limited detecting targets (7).

Plenty of drugs have been used in the treatment of *B. gibsoni* infection, including the common antiprotozoal drug diminazene aceturate (DA), acriflavine (ACF), and imidocarb without a clarified target or mechanisms of action (12, 13). Based on known inhibitors against other protozoan systems, using atovaquone combined with azithromycin against *B. gibsoni* came about as a new treatment strategy (14). However, the cases of relapse of cured dogs with clinical disease suggest incomplete elimination of parasites and drug resistance against *B. gibsoni*, showing that the challenges and difficulties of novel anti-*Babesia* drug development still exist (12, 13, 15). Hence, the mechanisms of commonly used drugs need to be elucidated, and validating a novel-specific inhibitor on a genome-wide scale is necessary.

For now, two canine babesiosis vaccines, Pirodog and Nobivac Piro, are commercially available (16). Aiming at canine babesiosis, soluble parasite antigens (SPAs) were prepared from culture supernatant to only protect dogs from B. canis or B. rossi (16, 17). A recombinant vaccine was developed against B. canis as well, meaning no vaccine is available for *B. gibsoni* (18). To develop a specialized vaccine against *B. gibsoni* or get wider cross-protection, whole-genome data are a well-developed platform in the search for vaccine-candidate antigens (19).

Thus, full-scale genomic data clearly provide great promise for improving diagnostic markers, drug target screening, and vaccine development (20). Over the years, whole-genome sequencing of apicomplexan has been promoted in an efficient and thorough manner with the establishment of next-generation sequencing (NGS) and third-generation sequencing (TGS), which are also known as high-throughput sequencing (HTS) and represented by PacBio, respectively (21, 22). The first genome of an apicomplexan, Plasmodium falciparum clone 3D7, was sequenced in 2002, revealing 14 chromosomes and about 5,300 encoding genes (23). Then, genome data of some *Cryptosporidium*, *Theileria* species, and more *Plasmodium* spp. were released (24–27). Genome sequencing of Toxoplasma gondii was done in 2009, and genome data are constantly updated due to its relatively large genomes and the flourishing of genomic study (28, 29).

For *Babesia*, the genome research began with sequencing the full genome of Babesia bovis in 2007, indicating four chromosomes and revealing a similar genome size to *Theileria* (30). From then on, a greater understanding of *B. bovis* in metabolism and drug design was introduced to a various range of babesial research (30). Next, the raw genome data of Babesia microti were assembled and annotated (31, 32). The genome data of Babesia ovata and Babesia bigemina were then available in the years that followed (33, 34). As yet, the whole-genome sequencing of *B. bovis*, B. bigemina, B. divergens, B. microti, *B. ovata*, B. canis, *Babesia* sp. *Xinjiang*, B. ovis, B. caballi, and B. duncani were released (34–36), and chromosome-level genome sequences are only available for *B. bovis*, B. canis, *B. bigemina*, B. microti, and *B. duncani*, leaving a large genome research space in extensive *Babesia* species.

Comparing *B. gibsoni* to *Plasmodium*, *Toxoplasma*, and previously stated *Babesia* species, only a small number of *B. gibsoni* genes have been identified and reported, and whole-genome analysis is not yet available, which results in a lack of full-scale genomic research (8, 37). Hence, a genome-wide scan of *B. gibsoni* is around the corner. The whole-genome sequencing of this hemiparasite not only serves as a solid foundation for genomic resource integration research on gene structure, karyotype analysis, and protein function, but it also provides instructive guiding of the entire life cycle activity of babesial parasites, including invasion, cell metabolism, host cell modification, and reproduction process, thus improving diagnostic markers, novel drug developments, as well as the *B. gibsoni* vaccine research (12, 14, 38, 39).

In this study, we sequenced and assembled the entire genome of *B. gibsoni* from apicoplast and mitochondria to the chromosomal genome. Genome maps were created using sequence data to obtain phylogenetic and colinearity relationships. Predictable protein secondary structure was achieved, and protein-protein interaction become precise. More scientific issues only observed on the genomic view are waiting to be tackled.

## RESULTS

### High-quality DNA samples.

**FIG 1:**
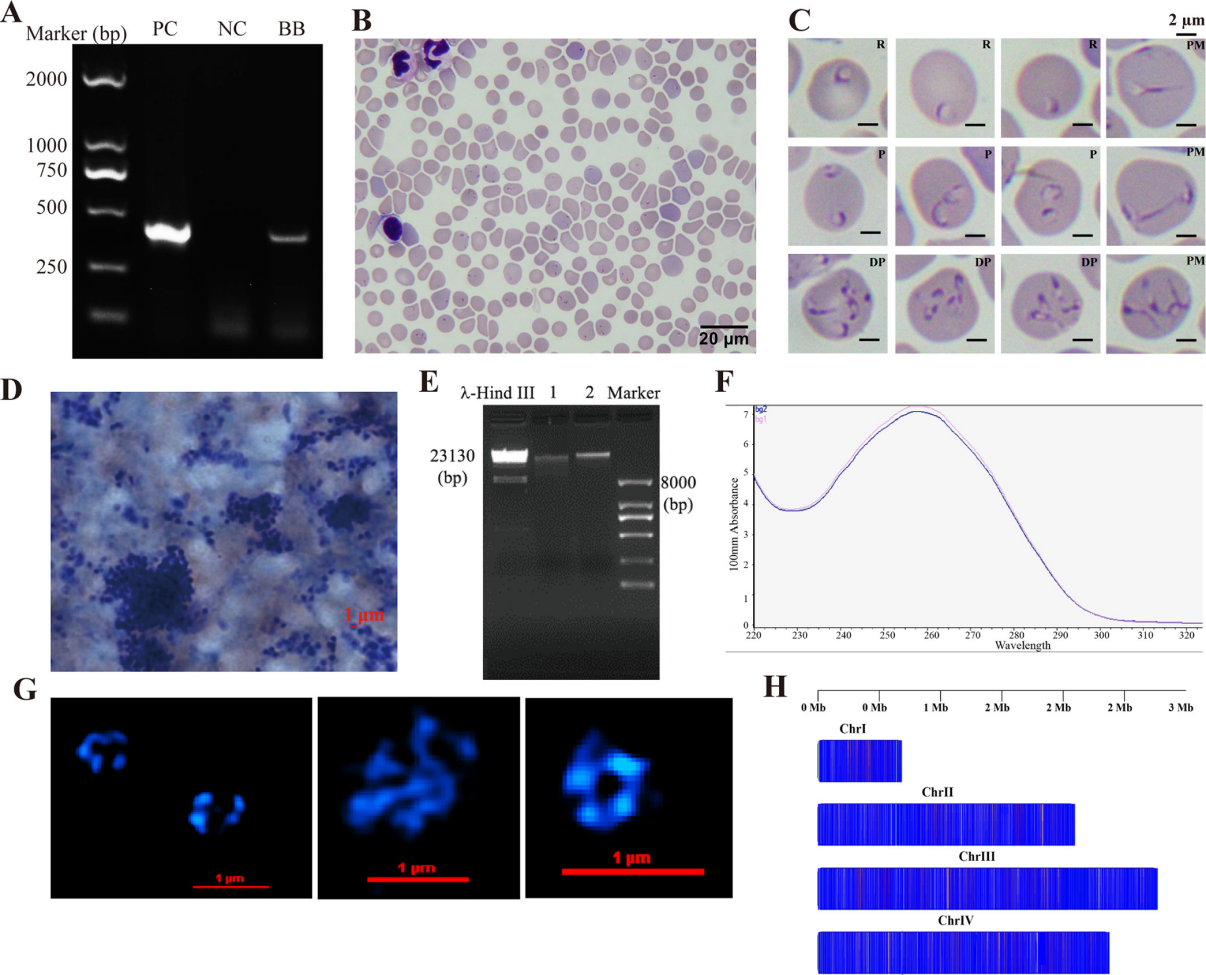
*B. gibsoni* purification and gDNA extraction. (A) PCR detection in experimental dogs. PC, Bg gDNA-positive control; NC, negative control; BD, blood sample of dog A. (B) Giemsa-stained blood smear from infected dog A. (C) Diversity of parasite forms. R, ring-shape merozoites; P, paired merozoites; DP, double paired merozoites; PM, pleomorphic merozoites. (D) Microscopic morphology of the parasite after merozoites collection. Merozoites of *B. gibsoni* was 1 to 2 μm in diameter. (E) Agarose electrophoresis of DNA. 1, Parasite DNA sample 1; 2, parasite DNA sample 2. For third-generation sequencing, the main band was clear and ≥23 Kb, indicating that the parasite DNA was not degraded and the sample was complete. (F) DNA detection of *B. gibsoni* by NanoDrop. (G) Chromosomes observed at different angles. (H) Chromosome pattern with sequencing size of *B. gibsoni*.

Parasitemia of the experimentally infected beagles was monitored by blood smears every day. Blood was collected when the proportion of parasitized erythrocytes (PPE) reached 20% at 30 days postinnoculation, and PCR detection was performed (Fig. 1A to C). It has always been a challenge to purify *Babesia* from infected animals without host leukocytes. In this study, 2-μm and 1-μm membrane filtration was first applied to remove host leukocytes, respectively. The isolated parasites were stained with Giemsa and then viewed by microscope until no dog leukocyte was observed. (Fig. 1D). Genomic DNA (gDNA) was extracted, purified, and subsequently subjected to samples by measuring concentration, degradation level, contamination, and purity. Table S1 (see supplemental material) shows that gDNA possessed a high level of purity and concentration. Acceptable DNA integrity with a visible single band during agarose electrophoresis (AGE) (Fig. 1E), as well as reasonable optical density at 260/280 nm (OD_260/280_) (Fig. 1F), suggested the final gDNA sample was available for genome sequencing.

### *B. gibsoni* sequencing and assembly.

Raw reads of *B. gibsoni* genome were obtained by Illumina Hiseq 2500 and PacBio. The assembled sequences, obtained from the original report, were present as six contigs, displaying high quality with an *N*_50_ length of 8.02 Mbp. The tig00000168 (51.9. Kbp) was removed from the nuclear genome for which it had a 99.9% similarity to the apicoplast genome (GenBank: MN481613.1). Then, another 5.9-Kbp mitochondrial genome (GenBank: KP666169.1) was confirmed and excluded from the chromosome component. The sequence of tig00000044 was found most partially overlapped with tig00000000 (93%) during a further comparison between five contigs by BLAST, and hence, they were merged into the same contig (tig00000000). As a result, a nuclear genome of 7,937,104 bp (7.94 Mb) in size and consisting of four contigs (tig00000000 tig00000002, tig00000017, and tig00000095) was confirmed. The number of chromosomes was confirmed by structure illumination microscopy that four distinct blue fluorescence clusters were observed (Fig. 1G) and suggested *B. gibsoni* has four chromosomes, which is consistent with other *Babesia* species. The sizes of four chromosome were 0.69 Mbp (chromosome 1: BgChrI), 2.10 Mbp (chromosome 2: BgChrII), 2.77 Mbp (=chromosome 3: BgChrIII), and 2.38 Mbp (chromosome 4: BgChrIV), respectively (Table 1).

**TABLE 1:** Chromosome size (Mbp) comparison between three *Babesia* species

Chromosome	*B. gibsoni* (WH58)	B. microti (RI)	*B. bovis* (T2Bo)
Chromosome 1	0.69	1.88	1.25
Chromosome 2	2.10	1.85	1.73
Chromosome 3	2.77	1.58	2.59
Chromosome 4	2.38	1.38	2.62

**FIG 2:**
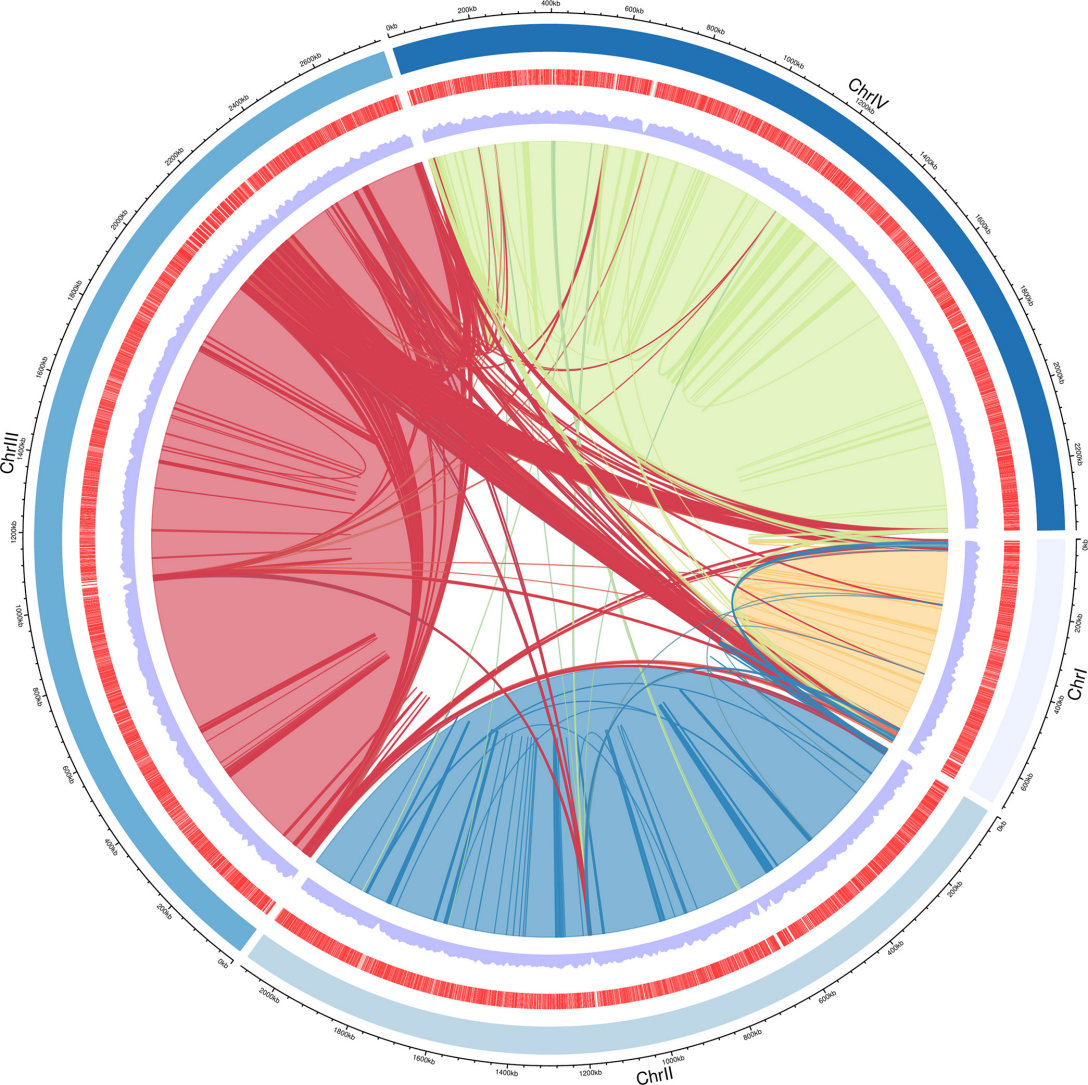
Circos display of the nuclear genome of *B. gibsoni*. The perimeter of the circos was divided according to chromosome size and colored with 4 different shades of blue, representing ChrI, ChrII, ChrIII, and ChrIV, from shallow to deep. Open reading frame of predicted 3,373 genes is shown as split orange strips. Gene density is represented by lilac wave band. A colinearity analysis of the chromosomes is the colored interior of the circos.

The genomic map, which was generated from OmicoStudio, showed a presentation of nuclear genomic composition and structure (Fig. 2). Chromosome length, the distribution of open reading frames, and gene density were divided into three layers for the schematic representation. A multicolored colinearity relation is marked in the central area.

### Genome annotation.

Prediction on protein-coding region, gene function, and noncoding RNA (tRNA, rRNA) was applied to genome annotation of *B. gibsoni* (Table 2). *Ab initio* data from Augustus and Genemark predicted 3,878 and 3,368 genes, respectively. Homology prediction from Exonerate generated 5,981 genes. RNA sequencing (RNA-seq) data were subjected to analysis by the hisat, stringtie, and transdecoder, revealing 2,786 protein-coding genes of annotation data. Eventually, a total of 3,552 complete genes were obtained by merging all export data from *ab initio*, homology, and RNA-seq by using the EvidenceModeler program (Table 2). The characterizations of the *B. gibsoni* genome were analyzed and compared with related apicomplexan parasites (Table 3).

**TABLE 2:** Statistics of annotated protein-coding genes

Evidence type	Programs	Total gene count
*Ab initio*	Augustus	3,878
	Genemark	3,368
Homology	Exonerate	5,981
RNA-seq	Hisat + stringtie + transdecoder	2,786
Merge	EVidenceModeler	3,552

**TABLE 3:** Comparison of genomic information of several common apicomplexan parasites

Genomic information	*B. gibsoni* (WH58)	*B. orientalis*	B. microti (RI)	*B. duncani*	*B. bovis* (T2Bo)	*Th. annulata* (Ankara)	P. falciparum (3D7)	Toxoplasma gondii
Total size (Mbp)	7.9	7.8	6.4	7.9	8.1	8.4	21.9	64.5
No. of chromosomes	4	4	4	4	4	4	14	12
Apicoplast size (Kbp)	28	33	29	34	33	NR^*a*^	35	35
Mitochondrion size (Kbp)	5.9	6.0	11.1	5.9	6.0	5.9	6.0	5.9
Total G + C composition (%)	44.1	42.4	36.3	37.7	41.6	32.5	19.3	52.2
5S_rRNA	4	NR	2	NR	9	3	3	47
18s_rRNA	2	NR	2	NR	3	NR	5	44
28S_rRNA	2	NR	2	NR	3	NR	6	32
tRNA	46	NR	44	NR	69	47	76	184
Protein coding genes	3,552	4,199	3,601	3,759	3,706	3,795	5,097	9,376

a NR, not reported.

The positions of rRNA and tRNA sequence of the nuclear genome were predicted by RNAmmer and tRNAscanSE 2.0, respectively. All rRNA of *B. gibsoni* located in ChrII (2 rRNAs) and ChrIV (6 rRNAs), covering four 5S rRNA, two 18S rRNA, and two 28S rRNA. Prediction of tRNA confirmed 8, 10, 12, and 16 tRNA sequences on BgChrI, BgChrII, BgChrIII, and BgChrIV, respectively. There is only one tRNA that has one intron located on ChrI while all the rest are intronless. All tRNA shared a mean GC content of 44.2%. Table S2 and S3 show more information about prediction results targeting RNA.

### KEGG/GO analysis.

KEGG pathway analysis and GO annotations were performed by using the NR database and visualized by the EGGNOG-MAPPER program (40, 41). A total of 2,641 putative proteins were annotated with functional information and enriched to 316 KEGG pathways (Fig. S2). The majority of functional annotations are enriched on genetic information processing (558 proteins), metabolism (453 proteins), and human diseases (395 proteins). Among genetic information processing, 97 translation-related and 86 transcription-related proteins were significantly enriched on the ribosome and spliceosome pathways, respectively. For metabolism, 176 proteins took up a large proportion of the terms of the global and overview maps. Among human diseases, 145 putative proteins were enriched to infectious diseases. More details are shown in Table S4.

A total of 2,627 genes were enriched to 608 GO categories at three terms, cellular component (CC), biological process (BP), and molecular function (MF) (Fig. S2). Significant amounts of annotations were distributed in BP (3,837 annotations) and CC (2,821 annotations) on the GO map. More than half of BP term annotations were enriched to cellular process (534 genes), metabolic process (506 genes), single-organism process (404 genes), and biological regulation (327 genes). Among the CC terms, cell (571 genes) and cell part (570 genes) were dominant annotations. MF class (913 annotations) showed salient terms on binding (409 genes) and catalytic activity (343 genes). Complete results are shown in Table S5.

### Synteny analysis.

**FIG 3:**
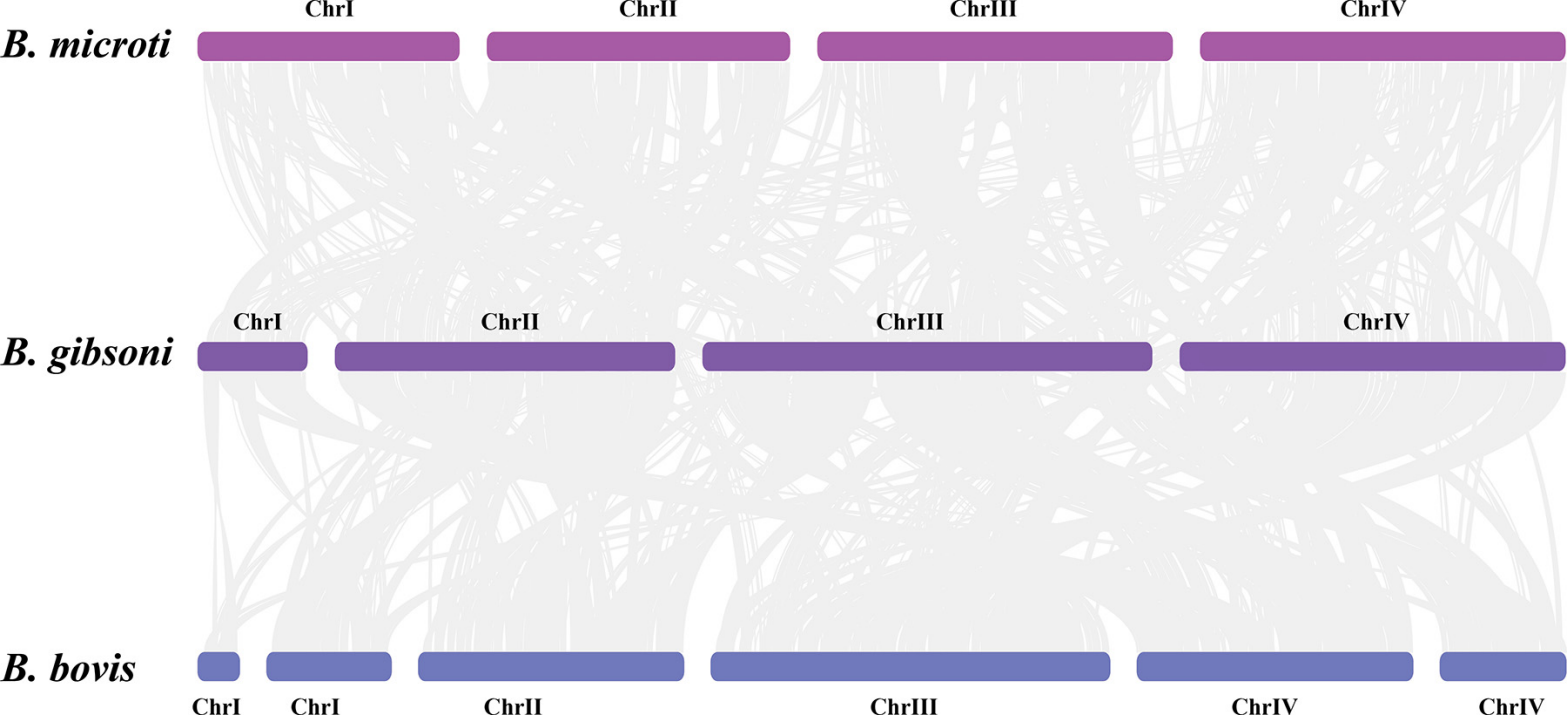
Whole map of nuclear genome synteny between *B. gibsoni*, *B. bovis,* and B. microti.

A homologous comparison was performed to conduct a genomic synteny analysis between *B. gibsoni*, *B. bovis,* and B. microti (Fig. 3). The incomplete annotation information of *B. bovis* was combined from all data online and added to the comparison system: contigs NW_001820853.1 and NW_001820854.1 of BbChrI, NC_010577.1 of BbChrII and BbChrIII, and NW_001820857.1 and NW_001820855.1 of BbChrIV. The annotation data of B. microti are thorough and complete; thus, NC_027205.1 (BmChrI), NC_027206.1 (BmChrII), NC_027207.2 (BmChrIII), and NC_034969.1 (BmChrIV) were added to the analysis system. For *B. gibsoni*, as stated above, tig00000095, tig00000017, tig00000000, and tig00000002 represented the four chromosomes from BgChrI to BgChrIV.

### New divergent timing point of *B. gibsoni*.

**FIG 4:**
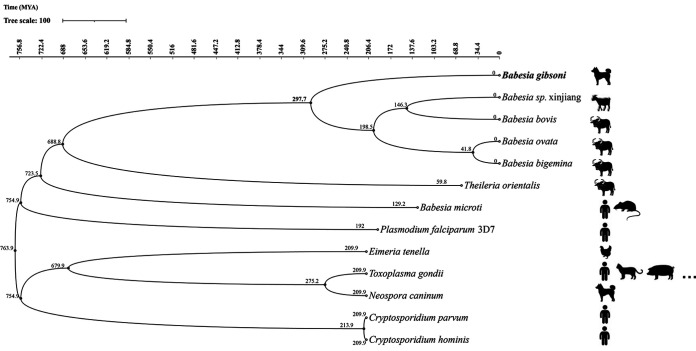
Species phylogenetic time tree analysis of the whole genome of *B. gibsoni* and other apicomplexan parasites.

A total of 65,266 genes from 13 apicomplexan parasites were considered for phylogenetic evolution analysis. OrthoFinder assigned 56,781 genes (87.0% of the total) to 9,312 orthogroups. Of these, 50% were in orthogroups with 10 or more genes, including the largest 2,198 orthogroups. Additionally, 881 orthogroups contained genes from all species, with 456 of these entirely single-copy genes. The phylogenetic file was output by OrthoFinder as a STAG species tree of the direct homologous group. Calculated by r8s, the divergent point of *B. gibsoni* occurred 297.7 million years ago, predating any other *Babesia* spp. except for B. microti, which diverged 34.7 million years ago earlier than *Theileria* spp. (Fig. 4).

### Venn diagram analysis among multiple apicomplexan members.

**FIG 5:**
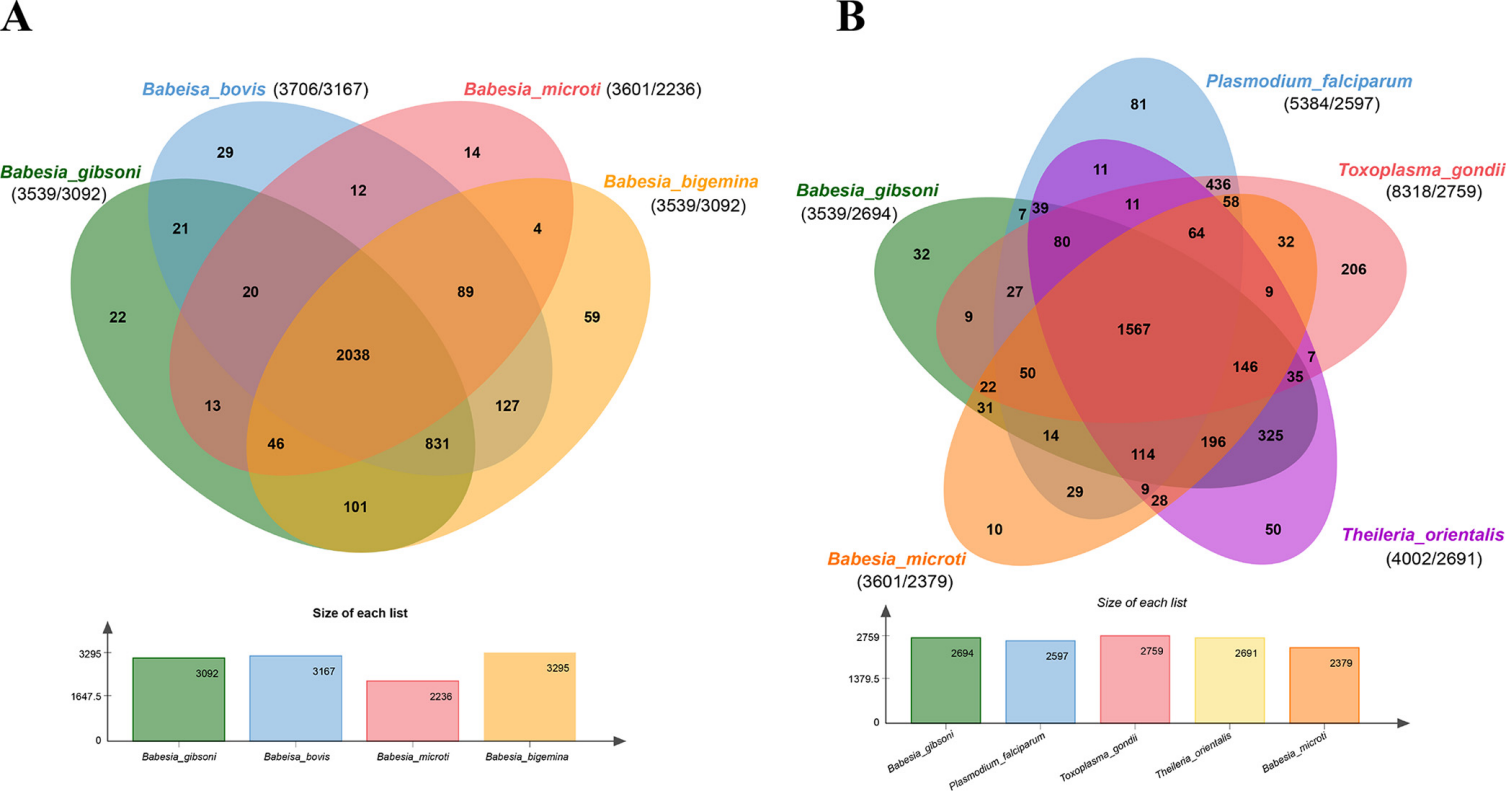
The Venn diagram of apicomplexan parasites genome sequences. (A) Comparison of orthologous clusters in four *Babesia* species. (B) Comparison between *B. gibsoni* and four apicomplexana species. The number of orthologous groups is indicated in the intersections. The total number of gene models and clusters for each species is shown in parentheses.

OrthoMCL was used to compare *B. gibsoni* with other related *Babesia* species, including *B. bovis*, B. microti, and *B. bigemina* (Fig. 5A). A total of 3092 annotated *B. gibsoni* protein-coding genes were aligned to orthologous groups, and 2,038 proteins of four *Babesia* species were shared as pan-*Babesia* conserved orthologous groups (COGSs). B. microti shared the least proteins that were apart from pan-*Babesia* clusters with three *Babesia* members. In addition, *B. gibsoni*, *B. bovis*, and *B. bigemina* held 831 other proteins in the same COG, which was significantly higher. A total of 22 unique proteins of *B. gibsoni* were functionally categorized with transporters (22.09%), specific secretory proteins (11.66%), hypothetical proteins (11.66%), merozoite proteins (8.59%), and surface proteins (3.68%).

The genomes of several apicomplexan parasites (*B. gibsoni*, P. falciparum, Toxoplasma gondii, *T. orientalis*, and B. microti) were cross-compared by OrthoMCL, respectively (Fig. 5B). The 5 parasites shared 1,567 lineal proteins of pan-apicomplexan parasites, which met the fundamental needs of parasites for survival. T. gondii owned 206 unique proteins, which was in line with its capacity for extensive host infection. In contrast, less distinction in genomic annotations among *Babesia* species (32 unique proteins of *B. gibsoni* and 10 unique proteins of B. microti) was deemed as a consequence of their narrow and highly specific host tendency and their specialized ecological niche.

### Metabolism pathway characterization.

**FIG 6:**
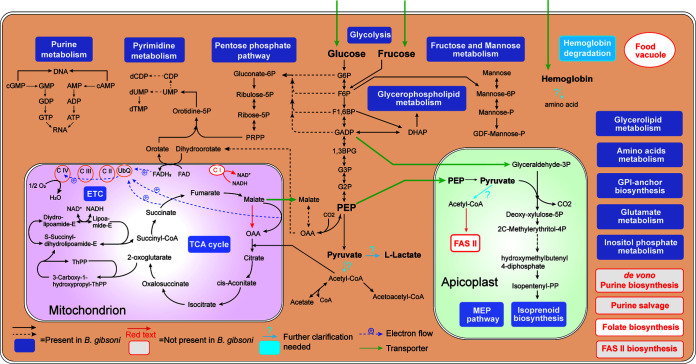
Map of major metabolism pathways in *B. gibsoni*. Solid arrows show the conversion of components within single step. Dashed arrows show complicated conversion with multiple omissions. (1) Metabolites: 1,3BPG, 1,3-bisphosphoglycerate; DHAP, dihydroxyacetone phosphate; F1,6BP, fructose 1,6-bisphosphate; F6P, fructose 6-phosphate; G2P, 2-phospholglycerate; G3P, 3-phospholglycerate; G6P, glucose 6-phosphate; GADP, glyceraldehyde 3-phosphate; OAA, oxaloacetate; PEP, phosphoenolpyruvate. (2) Pathways: ETC, electron transport chain; FAS II, type II fatty acid synthesis; MEP, methylerythritol 4-phosphate; TCA, citric acid cycle. (3) Enzymes: C I, complex I; CII, complex II; CIII, complex III; C IV, complex IV of the respiratory chain; UbQ, ubiquinone.

A metabolism pathway of *B. gibsoni* was mapped according to genome-wide annotation, involving in series of main routes that sustain the essential biological activities (Fig. 6).

Sugars such as glucose and fructose are obtained via transporter proteins, which then undergo glycolysis to produce pyruvate, and lactate is considered to be the main end product in central carbon metabolism (42). However, the annotation results revealed that *B. gibsoni* lacked lactic dehydrogenase (LDH). Using the *B. bovis* LDH gene locus as a reference, a synteny region was searched in *B. gibsoni*, and no gene was identified. Further transcriptome analysis only suggested the presence of canine LDH. Additionally, the absence of malate dehydrogenase (MDH), which was thought to originate from LDH activity (43), suggested a huge gap in *B. gibsoni* LDH. By implication, the enzyme responsible for catalyzing pyruvate to lactate of *B. gibsoni* still remains unknown.

At the end of glycolysis, the pyruvate is usually converted to acetyl-CoA by the pyruvate dehydrogenase (PDH) complex, implying its essential role in bridging the cytoplasmic glycolysis to the TCA cycle. However, it is a missing link in the apicomplexan mitochondrion, and apicoplast is responsible for all PDH complexes (44). Alternatively, branched-chain ketoacid dehydrogenase (BCKDH) was previously confirmed to have PDH activity in *Plasmodium* and *Toxoplasma* mitochondrion (45). In *B. gibsoni*, the PDH complex was completely absent, but the subunits or proteins of the constituent BCKDH complex were annotated, including BCKDH E1 alpha/beta and BCKDH E2. The specific role of the BCKDH complex in catalyzing the mitochondrial conversion of pyruvate to acetyl-CoA needs to be further verified.

The TCA cycle plays an essential role in energy production for *B. gibsoni*. As stated above, MDH was not identified, leading to the fact that malate could only be oxidized to oxaloacetate via malate-quinone oxidoreductase (MQO) in the mitochondrion, accompanied by the generation of electrons and then entering ubiquinone (UBq) reduction and electron transport chain. Full-scale sequencing of *B. gibsoni* also confirmed the apicomplexan-wide loss of complex I, termed NADH dehydrogenase. An NADH dehydrogenase-like conserved domain was predicted in *B. gibsoni* genome, indicating its function in generating NAD(P)H.

Hemoglobin degradation is an essential vital activity for *B. gibsoni* to obtain nutrients from host cells (46). Although heme detoxification protein (HDP) and falcipain 2, which were previously identified as complex components that function in *Plasmodium* hemozoin formation (47), were both annotated in *B. gibsoni* genome, no evidence of heme biosynthesis was given in *B. gibsoni*. In addition, the *B. gibsoni* lacked food vacuoles (46), the structural basis for hemoglobin degradation, indicating that the way *B. gibsoni* utilizes hemoglobin still leaves extensive gaps.

As an obligate intracellular protozoan, *B. gibsoni* possesses a minimal-size genome, resulting in a massive reduction in metabolomic compounds and therefore loss of key proteins involved in multiple metabolic pathways. The type II fatty acid synthesis (FAS II) pathway, which is an important *de novo* synthesis that has been clearly identified in *Plasmodium* and *Toxoplasma* apicoplasts (48), was found missing in *B. gibsoni* and piroplasms. Contrary to *de novo* pyrimidine synthesis, all apicomplexans are incapable of producing purine *de novo* (49), making purine salvage become an essential step in the purine compounds obtaining process (44). Purine salvage, however, was often considered incomplete in *Babesia* due to the absence of key enzymes (30). For instance, hypoxanthine-guanine phosphoribosyl-transferase (HGPRT), responsible for catalyzing the conversion of guanine and hypoxanthine to monophosphate nucleoside, was found to be absent in *B. gibsoni*. Nevertheless, the existence of purine salvage-involved enzymes, such as adenylosuccinate synthetase (ASS), adenylosuccinate lyase (ASL), and fumarate hydratase (FH), proved that *B. gibsoni* possessed the potential of producing fumarate in the cytosol and converting fumarate to malate (44). Folate biosynthesis was also found to be nonexistent, with only an interconversion between folate, dihydrofolate (DHF), and tetrahydrofolate (THF) being present.

### Genome structure at chromosome ends and distribution of *ves1-like* genes.

The genome structure at the ends of *B. gibsoni* was similar to *B. bovis*, comprising a subtelomeric region and telomeric sequence repeats in the pattern of CCCTA. Between the telomeric regions and the subtelomeric protein-coding region, *B. gibsoni* possessed a shorter noncoding region than *B. bovis*, which was at a length of 0.5 to approximately 1.5 kbp. A common feature of no repeats in this region was further confirmed, in contrast to two repeat regions in *Theileria* (50). Equally important, the first protein-coding genes in *B. gibsoni* occupied closer proximities to telomeres due to the shorter noncoding regions.

**FIG 7:**
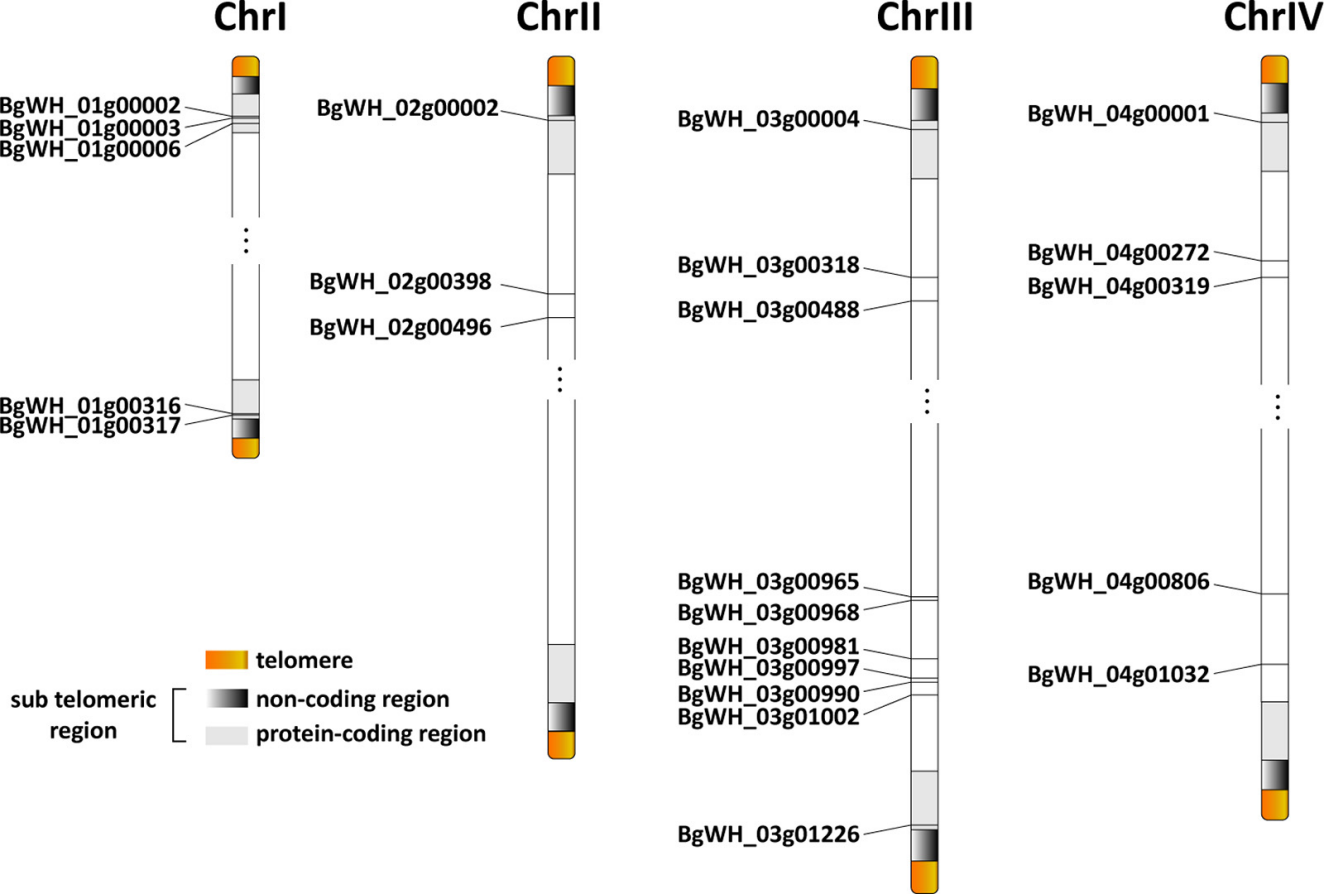
Distribution display of 23 members from *ves1*-like multigene family on 4 chromosomes.

The subtelomeric and telomeric regions of the *Plasmodium* chromosomal ends are crucial for preserving antigenic diversity (50). Contrarily, the largest *B. bovis* multigene family variant erythrocyte surface antigen-1 (*ves1*) is distributed all throughout the chromosomes, instead of merely at two ends (30, 50). Despite that the *ves1* gene family has not yet been clearly identified in *B. gibsoni*, 23 *ves1*-like genes were predicted in this study (Fig. 7). Nine of them (39.1%) were found in subtelomeric regions, with the remaining *ves1*-like genes dispersed across the chromosomes. Considering the extremely short length of the subtelomeric region, the *ves1*-like gene was denser in the region.

### Species-specific secreted antigen proteins.

*Babesia* species secrete a class of antigens to the host erythrocyte or supernatant while being cultured *in vitro*. These antigens either directly or indirectly affect the adhesion or invasion of the parasite to the host cell (51, 52), indicating a highly significant mechanism of physiological processes. A total of 3,572 proteins were predicted based on the signal peptide, transmembrane domain, and localization information, respectively (Table 4).

**TABLE 4:** Secreted proteins screened based on the genome-wide information of *B. gibsoni*

Program or db	Characteristic	The no. and proportion of possible secreted proteins on genome
Whole-genome annotation	All predicted protein	3,552 (100%)
SignaIP v5.0	Proteins contained signal peptide	428 (12.05%)
ProtComp v9.0	Extracellular protein	414 (113 extracellular + 301 no significance) (11.66%)
TMHMM v2.0 Phobius	Lack transmembrane domain or 1 transmembrane domain	376 (10.59%)

**FIG 8:**
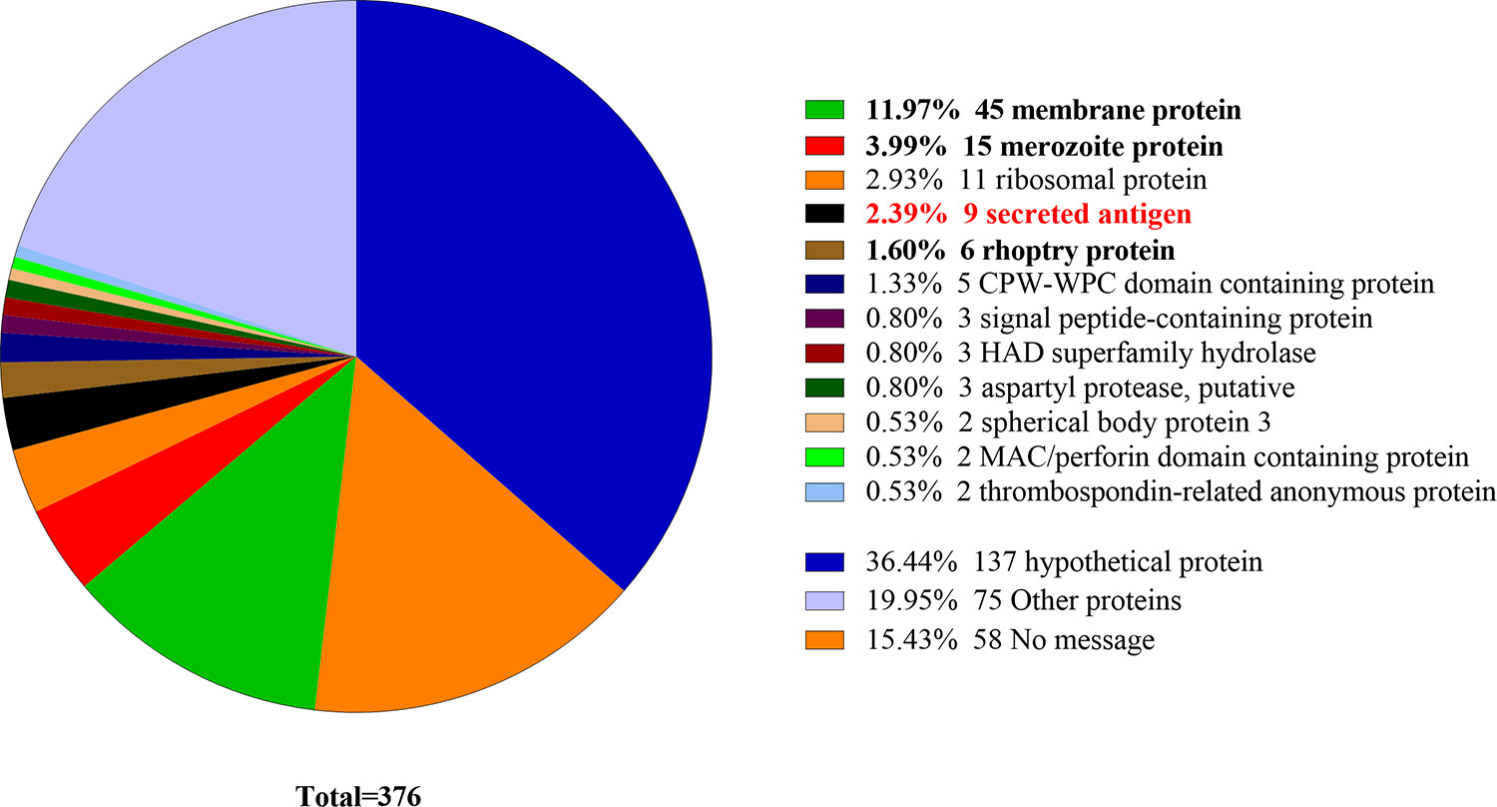
Distribution scale map of 376 putative secreted proteins. Despite 137 hypothetical proteins (navy blue), 75 other proteins (lilac), and 58 no message (orange), the 106 annotated proteins were classified by confirmed functions.

The output from SignaIP v5.0 revealed that 428 proteins of *B. gibsoni* possessed signal peptide domains, with 414 proteins among them localized in extracellular (113 proteins) or multiple locations (301 proteins with no significance). The transmembrane domain prediction of 414 proteins suggested that a total of 376 proteins (10.59%) were screened as putative secreted proteins, indicating 1 or no transmembrane domain. The majority of these putative secreted proteins have been found as invasion/adhesion associated by BLASTP (53), including 58 proteins with 137 hypothetical proteins that remained function unknown (36.44%), 45 membrane proteins (11.97%), 15 merozoite proteins (3.99%), and 9 secreted antigens (SA; 2.39%) (Fig. 8).

The screened 376 putative secreted proteins were analyzed with supernatant data *in vitro* by mass spectrometry, and 6 shared proteins were obtained and annotated. Five of them were annotated as T-cell immunomodulatory-like proteins, rhoptry-associated proteins, and rhoptry neck protein 2. BgWH_03g00242 was performed BLASTP comparison without any characterized protein. BgWH_03g00237 was aligned to secreted antigen 1 (fragment). Both BgWH_03g00242 and BgWH_03g00237 contained a signal peptide, a glycosylphosphatidylinositol (GPI) anchor site. Nevertheless, two distinct domains of coiled-coil (CC) and disorder were additional to BgWH_03g00237. Hence, a further comparison was performed on BgWH_03g00237 (shown in Table S6), and more 19 putative proteins were found to be homologous to BgWH_03g00237, revealing a phenomenon of high homology of amino acid sequence repeat at high frequency in species. Therefore, BgWH_03g00237 and the 19 putative proteins were characterized as the same protein family (Table 5). Fourteen putative proteins are annotated as secreted antigen 1 (SA1), and six of them are secreted antigen 3 (SA3). The structural domain of the 20 putative proteins was predicted, showing common information of 8 proteins (BgWH_03g00234, BgWH_03g00235, BgWH_03g00236, BgWH_02g00158, BgWH_02g00159, BgWH_02g00160, BgWH_02g00161, and BgWH_04g00735) on GPI sites, intrinsically disordered proteins (IDPs), and CC structure, which suggested that BgWH_03g00237 (GPI1) and eight more members (GPI2-9) of the GPI protein family were confirmed in *B. gibsoni* genome.

**TABLE 5:** The 20 selected proteins were annotated as the domain prediction of secreted antigen

No.	Gene ID	Disorder size	Colied-coil	Transmembrane and signal peptide	GPI^*a*^
1	BgWH_03g00237	154 aa	1	SP	+
2	BgWH_03g00238	11 aa		TM	−
3	BgWH_03g00235	61 aa	1	SP, TM	+
4	BgWH_03g00236	61 aa	1	SP, TM	+
5	BgWH_02g00159	284 aa	1	SP, TM	+
6	BgWH_03g00234	71 aa		SP	+
7	BgWH_02g00158	260 aa	1	SP	+
8	BgWH_02g00161	298 aa	1	SP	+
9	BgWH_02g00160	219 aa		SP, TM	+
10	BgWH_04g00735	138 aa		SP, TM	+
11	BgWH_03g00212	33 aa			+
12	BgWH_03g00809	28 aa	1	TM	+
13	BgWH_01g00258	30 aa			+
14	BgWH_04g00040				−
15	BgWH_04g00510			TM	−
16	BgWH_03g001229	11 aa		TM	+
17	BgWH_03g001228				+
18	BgWH_04g00039				−
19	BgWH_03g00130				+
20	BgWH_03g00003	38 + 17 aa			

a GPI, glycosylphosphatidylinositol; TM, transmembrane; SP, signal peptide; aa, amino acid.

### Transcriptions factors AP2 of sexual stage.

The apicomplexan apetala 2 gene family (ApiAP2), transcriptions factor encoding genes that were previously identified in the *B. bovis* genome, has been illustrated to regulate the developmental and life-cycling transitions during the asexual stage (54, 55). In combination with 20 members of ApiAP2 proteins (Table 6), they were construed that conserved hypothetical proteins in *B. gibsoni* genome comprise highly conserved domains made up of several β-fold and α-helix structures.

**TABLE 6:** The predicted ApiAP2 family members in *B. gibsoni* genome

No.	Gene ID	Accession	E value	Score	Description of target	Direction
1	BgWH_03g01047	PF00847.21	9.10E-41	137.9	AP2 domain	−^*a*^
2	BgWH_04g00976	PF00847.21	2.90E-17	62.7	AP2 domain	−
3	BgWH_04g00180	PF00847.21	3.50E-15	56	AP2 domain	−
4	BgWH_02g00573	PF00847.21	2.70E-13	49.9	AP2 domain	−
5	BgWH_02g00818	PF00847.21	3.20E-13	49.7	AP2 domain	−
6	BgWH_04g00046	PF00847.21	7.80E-12	45.3	AP2 domain	−
7	BgWH_04g01044	PF00847.21	3.60E-11	43.1	AP2 domain	−
8	BgWH_02g00392	PF00847.21	1.10E-10	41.6	AP2 domain	−
9	BgWH_04g00636	PF00847.21	2.90E-10	40.3	AP2 domain	−
10	BgWH_04g00729	PF00847.21	6.00E-10	39.2	AP2 domain	−
11	BgWH_01g00121	PF00847.21	2.10E-09	37.5	AP2 domain	−
12	BgWH_03g00908	PF00847.21	5.10E-09	36.3	AP2 domain	−
13	BgWH_04g00699	PF00847.21	6.10E-09	36	AP2 domain	−
14	BgWH_03g00279	PF00847.21	2.50E-08	34	AP2 domain	−
15	BgWH_02g00672	PF00847.21	3.00E-08	33.8	AP2 domain	−
16	BgWH_04g00240	PF00847.21	1.40E-07	31.7	AP2 domain	−
17	BgWH_04g00415	PF00847.21	2.50E-07	30.9	AP2 domain	−
18	BgWH_02g00293	PF00847.21	7.10E-07	29.4	AP2 domain	−
19	BgWH_03g00922	PF00847.21	3.90E-06	27	AP2 domain	−
20	BgWH_03g00432	PF00847.21	4.30E-06	26.9	AP2 domain	−

a According to the assembly results, the transcriptions of Bg AP2 genes start on the negative strand.

### Multiple invasion-associated proteins revealed complexity of mechanism.

Previously, apical membrane antigen 1 (AMA-1) and the rhoptry-associated protein (RAP) family were acknowledged to be present in *B. gibsoni* (56, 57), providing the foundation for the study of the invasion mechanism. The AMA-1 and RON proteins, which form a moving junction (MJ) and enable invasion in Toxoplasma gondii and *Plasmodium* spp., are expected to function as antigen-binding sites of vaccines (58, 59). Using multiple prediction tools, BgRON2 was first reported, together with the secondary structure and molecular docking feature between BgAMA-1, and the BgRAPs pattern of distribution was revealed.

The microneme protein AMA-1 of *Babesia* plays significant roles during the moving junction process and internalization, which indicates it is a powerful candidate for drug targets or diagnostic markers. BgAMA-1 is highly conserved, which is in accordance with current research, yet more faithful nucleotide sequences of the encoding genes are determined to be located on chromosome ChrIII. The conserved domains of BgAMA1 include signal peptide, extracellular domain, transmembrane domain, and intracellular domain. The P3AMA region of BbAMA-1, which showed the highest sensitivity against neutralizing antibodies (58), is aligned to a conserved region of BgAMA-1 with an identity of 79.3%, increasing the likelihood that BgAMA-1 will serve as a prospective epitope.

**FIG 9:**
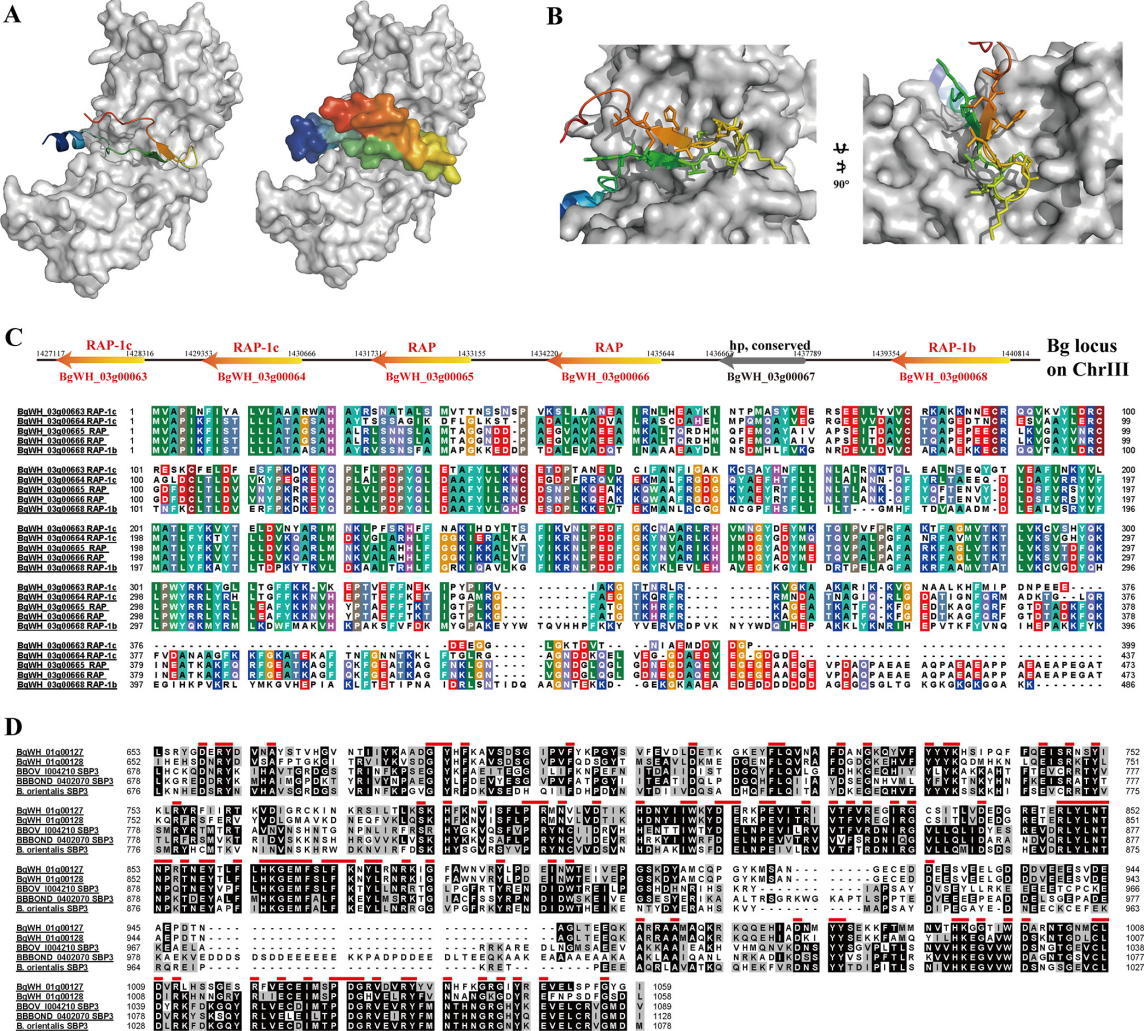
The modeling and secondary pattern of multiple invasion-associated proteins. (A) A docking model of BgAMA1 (gray, sphere texture) and BgRON2-docking-region (E1238-V1273 were multiple-colored, cartoon texture). (B) Predicted docking model between BgAMA1 and BgRON2 with full sphere texture and a zooming focus on docking region. (C) A tandemly arranged pattern of BgRAPs on ChrIII. (D) Two forms of Bg SBP3 proteins aligned to *B. bovis* and *B. orientalis* SBP3; identical amino acids were highlighted in red lines.

Another moving junction involved and host-cell targeted protein, rhoptry neck protein 2 (RON2), was predicted as well. The disulfide-bound-hairpin loop was the key to molecular interactions while complex formation and extra residues of *PfRON2sp1* located in the N and C termini directly point to a higher affinity between AMA-1 and RON2 (59, 60). Highly consistent with *Plasmodium*, a function-like binding domain predicted that a most divergent region called cystine loop, which was involved in the formation of negligible buried surface area and species selectivity, is incorporated within BgRON2 (residues E1238-T1275) (Fig. 9A and B).

All of the *Babesia* species that have been studied thus far have been shown to contain the RAP encoding genes (61). The RAP encoding genes in the *B. gibsoni* genome were distributed as many gene families that were tandemly organized on ChrIII and have a high identity between six proteins. BgWH 03g00663 and BgWH 03g00664 are both labeled as RAP-1c and shared a 49.2% identity. In accordance with *B. bovis*, *B. gibsoni* possesses two copies of the *rap* gene adjacent to each other that encode two same RAPs (BgWH_03g00665 and BgWH_03g00666) (Fig. 9C).

Spherical body proteins (SBPs) are specific proteins to *Babesia* spp. and *Theileria* spp., which are localized in the cytoplasm of erythrocytes and get involved in the secretion of apical organelles during the merozoite stage and lead to a unique mechanism of invasion (62, 63). Multiple sequence alignments were performed between *B. gibsoni* genome and SBPs (SBP2, SBP3, and SBP4) from *B. bovis.* Given the lack of evidence for the presence of SBP1, SBP2, and SBP4 in the *B. gibsoni* genome, only BgWH_01g00127 and BgWH_01g00128 were termed as SBP3 (Fig. 9D). Despite the fact that they were considered to be two forms of SBP3, both matched the molecular weight features of *B. bovis* SBP3, which were 123.4 kDa (BgWH_01g00127) and 122.1 kDa (BgWH_01g00128), respectively. Most significantly, BgWH_01g00127 and BgWH_01g00128 shared an identity of 63%, and sequence alignment showed that there was only one gap between these two proteins.

Another conserved rhoptry function associated protein PfCERLI1 was recently reported to be essential for rhoptry secretion and merozoite invasion of P. falciparum by three different lipid-interacting moieties: pleckstrin homology (PH) domain, C -domain, and an N-terminal alpha helix SP (64). A putative CERLI1-like protein, BgWH_04g00700, was first reported in this study that also owned a PH domain in the C terminus and a C2 domain, which had been confirmed to have a similar function of lipid-binding to the rhoptry membrane in P. falciparum. Of all the template information, the top three with the highest confidence (98.1, 97.6, and 97.5) and several dispersive prediction results with lower confidence all point to a C2 domain-like structure, which is annotated as calcium/lipid-binding domain (CaLB) that is derived from the PLC-like family. In addition, munc13-1, a Ca^2+^-phospholipid-dependent protein that contains multiple C2 domains as versatile protein-protein interaction modules, is further predicted (confidence 96.7). Thus, BgWH_04g00700 might function as a phospholipid-binding and metal-binding protein. In quick succession, another PH domain-like barrel is predicted with another high confidence value (97.3), which shared a similar structure to that a protein kinase B/Akt bound to protein membrane localization associated with inositol IP_4_. Both domains have the potential for close connections with rhoptry membrane binding.

### The vanished genes.

The minimal genome size of *B. gibsoni* is speculated to be the result of a particular, compulsory parasitism that uses a variety of tactics (61). During the research of *B. bovis*, merozoite-associated antigens (MSAs) have been demonstrated to have effective antigenicity (58, 65). Unexpectedly, the excellent potential target was found vanished from *B. gibsoni* genome. In addition, no homology of the abundant protein merozoite-associated protein (MSPs) of *Plasmodium* spp. during erythrocyte invasion was discovered in the *B. gibsoni* genome. Another gene family related to apicomplexan parasite antigenic variation is the *var* genes, which are manipulated by intricate molecular mechanisms and cause immune evasion, and they are once again less prominent in *B. gibsoni* (66, 67). Further research is required to determine whether these observations imply that *B. gibsoni* possesses particular invasion-related proteins and immune escape mechanisms.

## DISCUSSION

### Chromosome structure and evidence of gene rearrangement.

Piroplasm, including *B. gibsoni*, generally possesses significantly smaller genome size and chromosome number than *Plasmodium* and *Toxoplasma*, which suggested the diversity of apicomplexan genome size and structure may be associated with host range and life cycle. The protein-coding genes in *B. gibsoni* are also much less than in *Theileria*, *Toxoplasma,* and *Plasmodium*, indicating a host-specific adaptation, limited host range, and reduced metabolism pathway. Compared to reported Babesia genomes, for example, *B. bovis* and B. microti, *B. gibsoni* displayed an inhomogeneity in chromosome size. BgChrI was about one-third in size of the rest three chromosomes, and BgChrIII was apparently larger.

Although high similarity, good synteny, and being relatively conserved between three *Babesia* species on the genomic level were revealed by amino acid sequence alignment (Fig. 5), significant chromosomal rearrangements suggested the occurrences of some important events during the evolutionary process. *B. gibsoni* shared 2,903 and 1,865 genes of significant genomic synteny with *B. bovis* and B. microti, respectively, and only 1,713 genes with synteny were shared between B. microti and *B. bovis*, which indicated a maxim of relevance between *B. gibsoni* and *B. bovis*. A higher degree of synteny was found between *B. gibsoni* and B. microti in ChrII (BgChrII and BmChrII) and ChrIV (BgChrIV and BmChrIV). In contrast, the synteny of ChrI and ChrIII was found weakened. The synteny analysis revealed *B. gibsoni* underwent gene rearrangement and deletion events, which were speculated as the consequences of selection pressure under the specific environment of canine erythrocytes, vicissitudinous living conditions, or other circumstances, causing its distinct phenotype and unique disease resistance. Venn revealed that the diversity of encoding proteins was inversely proportional to parasite specialization level in various environments, ecological niches, and lifestyles, thus reflecting lineage-specific adaptations (4, 31, 68).

### SA proteins that modified with GPI provide options for drug targets.

Some secretory proteins can be obtained by mass spectrometry analysis of serum, secretions, or supernatant *in vitro* culture. However, none of these methods are applicable to low-abundance proteins (18, 52). From the genomic level, proteins with signal peptides at the N-terminal or transmembrane domains could be screened by whole-genome information of parasites and bioinformatic analysis, which is regardless of protein expression abundance and takes advantage of high throughput (69). In-depth research on apicomplexans has focused on the secreted proteins involved in adhesion and invasion, such as rhoptry, microneme, and dense granules. In addition, numerous secretory proteins collaborate with proteins that possess GPI sites, accelerating the process of adhesion and invasion (67, 70, 71). In *B. bovis*, the variable merozoite surface antigens (VMSA) have been considered an important candidate for vaccine development, which neutralizes parasites from the invasion process of erythrocytes by anti-VMSA proteins *in vitro* (72). Relating proteins modified with GPI have been reported to bind to the cell membrane to form complexes, and GPI anchors can be removed with phosphoacyl-specific phospholipase C (PI-PLC) to convert proteins into the soluble proteins and release them from the cell membrane surface into extracellular medium (73, 74). Since proteins with GPI anchor sites are exposed on the cell membrane’s surface or split from the cell membrane after parasites infiltrate the host cells, the host’s protective immunity could be potentially triggered and a viable target of anti-*B. gibsoni* medication.

### AP2 of sexual stage.

BgWH_03g01047 shared the highest degree of similarity of AP2 genes with *Babesia* and *Theileria equi (T. equi)*. Domains 2 and 3 of BgWH_03g01047 appeared to correlate with evolutionary relationships of AP2 domains (BBOV_I04850_D2/D3) between *B. bovis* and other pirolasma, which were labeled as a chromalveolate ancestor branch. The great levels of orthology between *B. gibsoni* and *B. bovis* led to the assumption that the domain was derived from chromalveolates (54). Several apicomplexan ApiAP2 genes have distinct DNA binding preferences. Unique AP2 sequence motifs of P. falciparum occurred from binding preferences and interactions with secondary motifs among 60 highly conserved domains (54). BgWH_02g00818 was found to possess common structures with *Plasmodium* (Pf14_0633). However, differing motifs indicated a unique DNA binding pattern of BgWH_02g00818.

In conclusion, the genome study of *B. gibsoni* aims at elucidating and illustrating all its genes and its biological function, including canine-specific invasion mechanism, metabolism pathway, cycle regulation, etc. Through whole-genome sequencing and annotation, new insights into genome structures, evolution procedures, and pathogenic mechanisms will be gained. A novel viewpoint on the regulation of virulence, antigenic variation, and immune escape will emerge. Gene investigation on *B. gibsoni* will bring new hope to biological marker detection, drug-resistant markers, new drug targets, and vaccine development.

## MATERIALS AND METHODS

### Parasite strain.

The *B. gibsoni* WH58 strain was initially isolated from a naturally infected dog in Wuhan, China. After 280 days of domestication, the strain was stably maintained *in vitro* with a culture cycle of 3 to 6 days. The isolate was cryopreserved in liquid nitrogen in the Department of Veterinary Medicine of Huazhong Agricultural University in Wuhan, China.

### DNA extractions and purifications.

*B. gibsoni* WH58 strain was rapidly thawed in 37°C thermostat water bath and used to intravenously inoculate two laboratory beagles acquired from Anlu Laboratory Animal Center. Daily rectal temperature, Giemsa staining of blood smears, PCR detection, and microscopic examination were carried out to monitor the PPE. After obtaining approval from the Scientific Ethics Committee of Huazhong Agricultural University (permit no. SYXK 2015-0084) and following the Animal Ethics Procedures and Guidelines of the People’s Republic of China, a modest amount of blood was collected from day 31 postinoculation with anticoagulant tubes containing EDTA-K2. The sample was centrifuged at 1,000 rpm for 10 min to separate the erythrocytes from the supernatant. The product was then lysed with red blood cell lysing reagent (a solution of 1.0 g KHCO_3_, 8.3 g NH_4_Cl, and 0.037 g EDTA-Na_2_ was prepared by adding double-distilled water to 1,000 mL) for 10 min, followed by centrifugation at 1,000 rpm for 10 min. The precipitation was subsequently filtered by a 1-μm filtration membrane to remove white blood cells and residual erythrocytes. The sample was resuspended in normal saline (NS) and centrifuged at 12,000 rpm for 10 min twice to obtain pure parasites in the precipitation. Finally, the product was suspended in an appropriate volume of NS and extracted by using TIANamp Genomic DNA kit (Qiagen, Shanghai, China). The *B. gibsoni* genome DNA (Bg gDNA) product was stored at −20°C for future use. The purity (OD_260/280_ and OD_260/230_), concentration, and nucleic acid absorption peaks of Bg gDNA were detected by Nanodrop. Qubit was used to obtain an accurate detection of gDNA concentration. Both the results were compared to assess the quality of the final product of gDNA. The integrity of gDNA was determined using the AGE method.

### RNA extraction and cDNA preparation.

RNA was extracted from 400 μL infected blood using the TRIzol method. A total of 800 μL of TRIzol was added to the sample in a RNase-free centrifuge tube and mixed by vortexing for 3 to 5 min. The sample was then allowed to stand at room temperature for 5 minutes to ensure complete lysis. The product was mixed with 200 μL chloroform and vigorously vortexed for 15 s and then incubated on ice for 5 min to allow for full phase separation. Next, the sample was centrifuged at 12,000 rpm and 4°C for 15 min. The sample was separated into three layers, with the top layer being a colorless aqueous phase, the middle layer a white flocculent layer, and the bottom layer a red organic phase. The top layer (400 μL) was transferred to a new RNase-free centrifuge tube, and an equal volume of isopropanol was added, mixed, and then precipitated for 30 min at 0°C or overnight at −20°C. After centrifugation at 12,000 rpm and 4°C for 15 min, the supernatant was discarded, and the RNA pellet was washed with 750 μL of ethanol and 250 μL of DEPC water and gently shaken at the bottom of the centrifuge tube and suspended. After centrifugation at 12,000 rpm and 4°C for 15 min, the supernatant was discarded and the pellet was dried at room temperature. The pellet was dissolved in 25 μL of sterile water, aliquoted, and stored at −80°C for later use.

The PrimeScript RT reagent kit with gDNA Eraser (TaKaRa, China) was used to perform reverse transcription on the extracted RNA. The gDNA Eraser reaction was setup on ice (5× 2 μL gDNA Eraser Buffer, 1 μL gDNA Eraser, 1.5 mL total RNA, and 5.5 μL DNase-free water) and incubated at 42°C for 5 min. The reverse transcription reaction (10 μL gDNA Eraser reaction product, 1 μL PrimeScript RT Enzyme 4 μL MixI, RT Primer Mix, 5× 4 μL PrimeScript Buffer2, and 1 μL DNase-free water) was then set up on ice and incubated at 37°C for 15 min followed by a 5-s incubation at 85°C. The cDNA was then stored at −80°C for future use.

### Synchronization for chromosome number determination.

To obtain high-resolution image with visible chromosomes, xanthurenic acid (XA) was employed to get plentiful parasites in the sexual stage. Four different levels of XA concentration (0 μM, 50 μM, 100 μM, and 200 μM) were applied to *B. gibsoni* WH58 strain *in vitro* cultures and, respectively, incubated at 28°C for 0 h, 3 h, 6 h, 12 h, 24 h, 32 h, 36 h, and 48 h to induce sexual stage. The induced parasites were cultured with 0.4 μg/mL colchicine for 6 h to maintain the synchronization status of gamogenenesis and then centrifuged at 1,000 rpm for 10 min. The pellet was resuspended with potassium chloride solution (0.075 mol/L) at 37°C for 15 to 20 min. The parasites in suspension were fixed by 3:1 of glacial acetic acid and methanol and then centrifuged at 1,000 rpm for 10 min and resuspended with 0.5 mL fixative. A thin blood smear was prepared by the final suspension and treated with Hoechst for 5 min, and then chromosomes were visible by structure illumination microscopy Nikon N-SIM.

### Genome sequencing and assembly.

The Illumina Hiseq 2500 and PacBio Sequel were both employed to obtain raw data. Initial contigs were assembled from raw NGS data and genome structures were constructed from TGS data. Contig-misassembly was then detected and corrected by CAP3 (75) and HGAP3 (76). Tandem repeats were analyzed on RepeatMasker 4.1.1 (77) based on Tandem Repeat Finder tool TRF version 4.0.9 (78) and RepBase (79).

### Gene annotation.

The prediction was first trained by using published *B. bovis* genome data as a model on Augustus v2.5.5 (80). *B. gibsoni* full-scale prediction was then accomplished by comparing homologous species sequences available from public databases and unreleased expression data of *B. gibsoni* from the transcriptome. Genome self-prediction was conducted using Genemark v2.5 (81). Homologous sequence alignment was obtained by nucleotide sequence alignment by using *B. gibsoni* data and five related species (*Babesia* sp. Xinjiang, *Babesia ovata*, Babesia bovis, Babesia microti, and Babesia bigemina) on Exonerate v2.2 (82). RNA-seq prediction and annotation results were merged from output files obtained by HISAT v0.1.3-beta (83), StringTie v1.0.4 (84), and Transcoder-Trinity v2.0.6 (85). The synteny analysis was performed on MCScanX (86). KEGG pathway enrichment and GO annotation were used to obtain functional annotations of genes by the NR database and EGGNOG-Mapper 5.0 (40, 41). Gene family clustering was predicted on orthoMCL (87). Single-copy genes were predicted based on multiple sequence alignment by MUSCLE (88). The noncoding RNA gene was annotated on tRNAscanSE 2.0 (89) and RNAmmer (90). The final results of whole-genome prediction were completed by integration on EVidenceModeler v1.1.1 (91) and BLAST tools (92). OmicStudio (93) was applied to generate a circos display of the assembled complement of chromosomes.

### Protein prediction.

Pfam (94) and TMpred (95) were, respectively, used to predict functional domains of specific genes and transmembrane regions. Secondary structure prediction based on homology modeling was performed on SWISS-MODEL Workspace (96). Protter version 1.0 (97), Phyre2 v2.0, and PyMOL (98) were visualization tools for domains and docking regions modeling.

### Phylogenetic tree construction.

Maximum likelihood reconstructions of phylogenetic trees of 13 apicomplexan protozoan species, including 6 *Babesia*, 2 *Cryptosporidium*, 1 *Theileria*, 1 *Plasmodium*, 1 *Eimeria*, 1 *Neospora*, and Toxoplasma gondii, were constructed by RaxML (99) and MEGA 7.0 (100). STAG species tree was generated from single-copied orthology searching results in OrthoFinder v2.5.1 (87) and r8s v1.81 (101) to calculate the divergence time of species in the evolutionary tree. The iTOL was used to visualize and manipulate the divergent point (102).

### Data availability.

The whole-genome sequence data of *B. gibsoni* WH58 strain has been deposited in the Genome Warehouse in the National Genomics Data Center, Beijing Institute of Genomics, Chinese Academy of Sciences/China National Center for Bioinformation, under accession number GWHBJTY00000000. Refseq of genomes used in this study (NCBI, https://www.ncbi.nlm.nih.gov): GCF_000165395.2 (*B. bovis* T2Bo), GCF_000691945.2 (B. microti RI), GCF_000981445.1 (*B. bigemina* BOND), GCF_000165365.1 (*T. parva* Muguna), GCF_000002765.5 (P. falciparum 3D7), GCF_000006565.2 (T. gondii ME49), and GCF_000740895.1 (*T. orientalis* Shitoku).
