# Supplementary material for: Babesia gibsoni Whole-Genome Sequencing, Assembling, Annotation, and Comparative Analysis
Source: Microbiol Spectr. 2023 Jul 11;11(4):e00721-23. doi: 10.1128/spectrum.00721-23 (PMC10434002; doi:10.1128/spectrum.00721-23)
Supplement: Supplemental file 1 — Figure S1. Download spectrum.00721-23-s0001.pdf, PDF file, 0.03 MB [file spectrum.00721-23-s0001.pdf]

Figure S1 Quality reports of full genome assembly.

Report

	Babesia_gibsoni	Babesia_bovis	Babesia_microti
# contigs (>= 0 bp)	6	14	6
# contigs (>= 1000 bp)	6	14	6
# contigs (>= 5000 bp)	6	13	6
# contigs (>= 10000 bp)	6	12	6
# contigs (>= 25000 bp)	6	8	5
# contigs (>= 50000 bp)	5	6	4
Total length (>= 0 bp)	8023965	8179706	6434485
Total length (>= 1000 bp)	8023965	8179706	6434485
Total length (>= 5000 bp)	8023965	8178557	6434485
Total length (>= 10000 bp)	8023965	8172552	6434485
Total length (>= 25000 bp)	8023965	8118796	6423938
Total length (>= 50000 bp)	7988960	8055423	6395281
# contigs	6	14	6
Largest contig	2772535	2593320	1816206
Total length	8023965	8179706	6434485
GC (%)	43.94	41.59	36.17
N50	2379944	1797577	1766409
N75	2098135	827912	1508385
L50	2	2	2
L75	3	4	3
# N's per 100 kbp	0.00	0.01	1.59

All statistics are based on contigs of size >= 500 bp, unless otherwise noted (e.g., "# contigs (>= 0 bp)" and "Total length (>= 0 bp)" include all contigs).

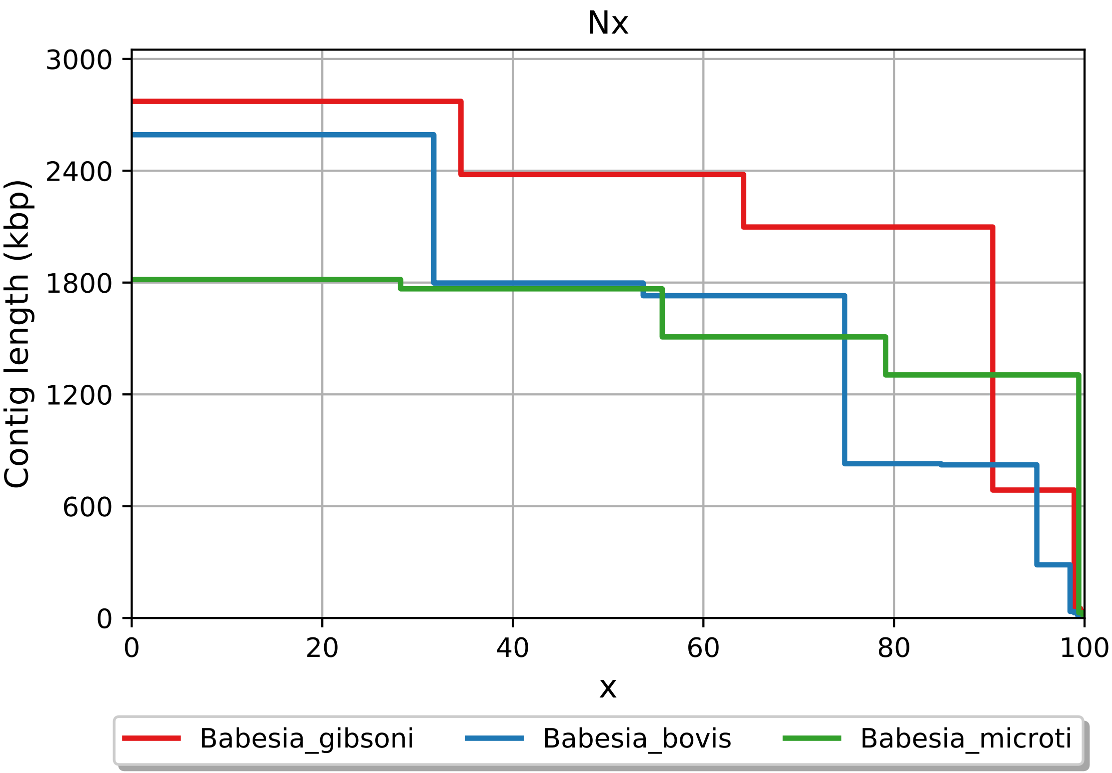

Nx
3000
2400
1800
1200
600
0
0
20
40
60
80
100
Contig length (kbp)
x
Babesia_gibsoni
Babesia_bovis
Babesia_microti

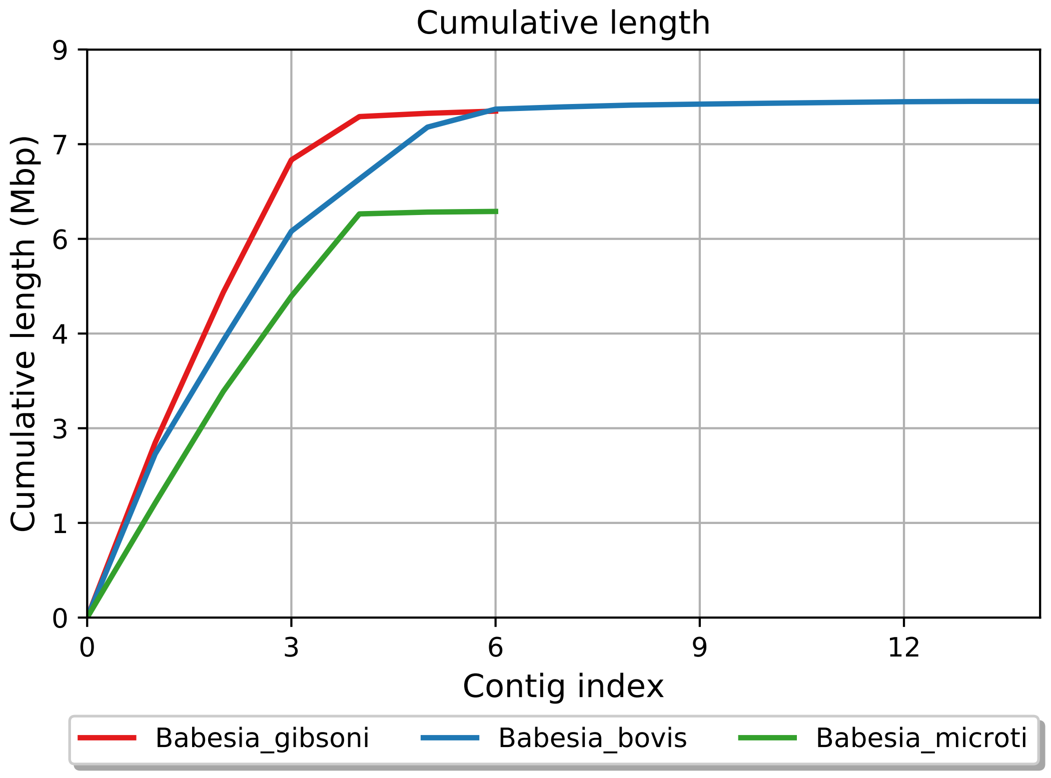
Cumulative length
Cumulative length (Mbp)
Contig index
0
1
3
4
6
7
9
0
3
6
9
12
Babesia_gibsoni
Babesia_bovis
Babesia_microti

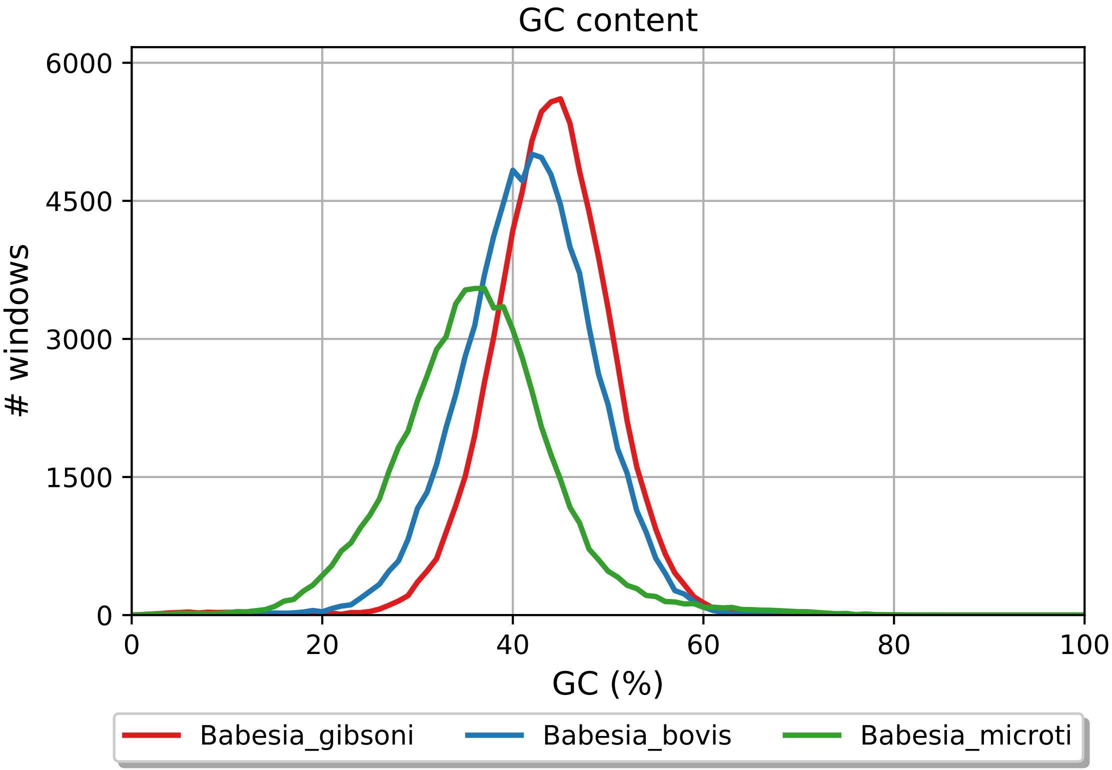

GC content
6000
4500
3000
1500
0
windows
0
20
40
60
80
100
GC (%)
Babesia_gibsoni
Babesia_bovis
Babesia_microti

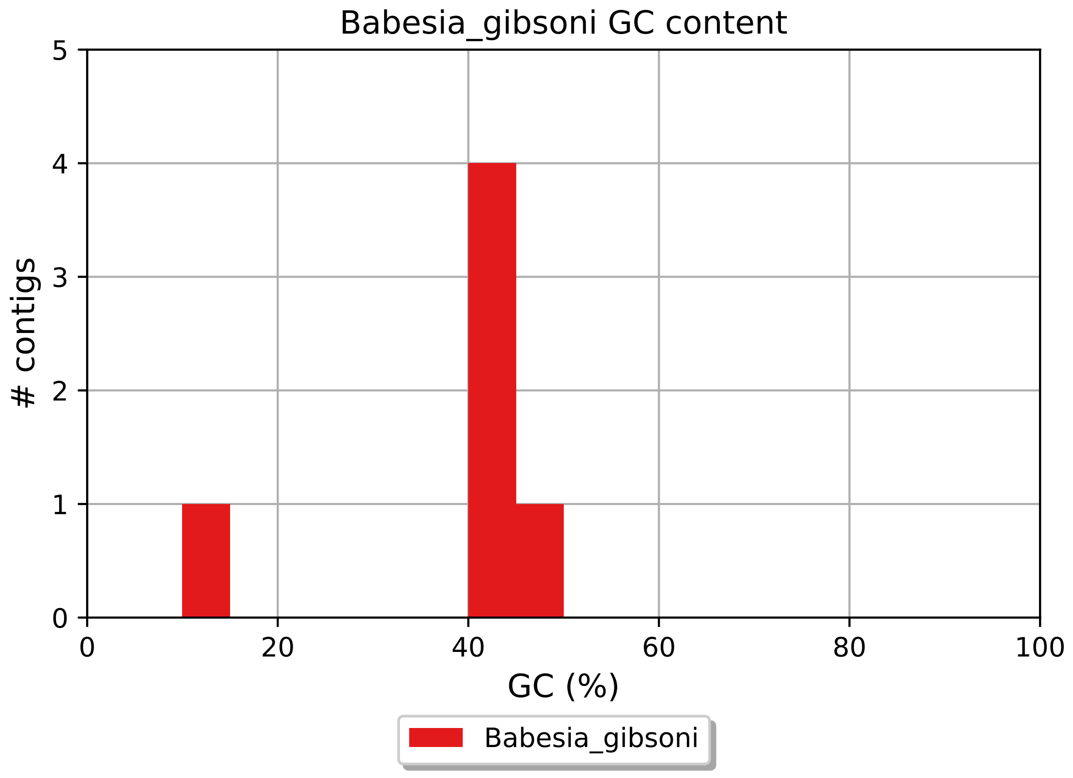

Babesia_gibsoni GC content
contigs
0
1
2
3
4
5
0
20
40
60
80
100
GC (%)
Babesia_gibsoni

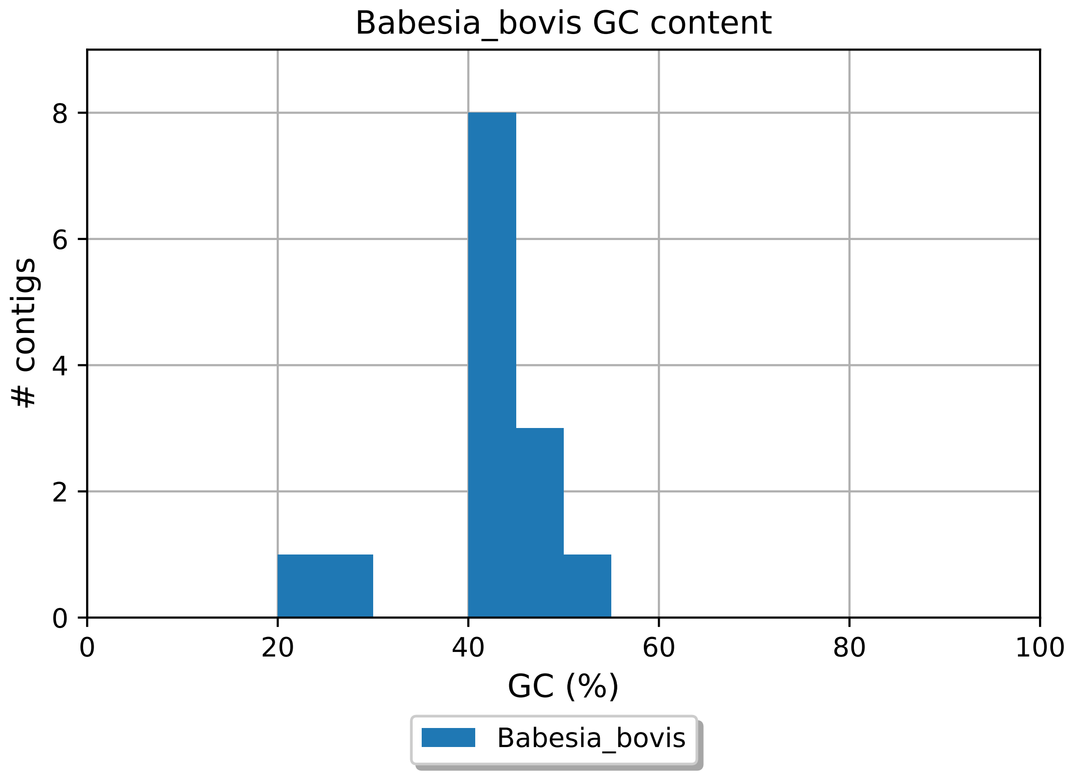
Babesia_bovis GC content
contigs
8
6
4
2
0
0
20
40
60
80
100
GC (%)
Babesia_bovis

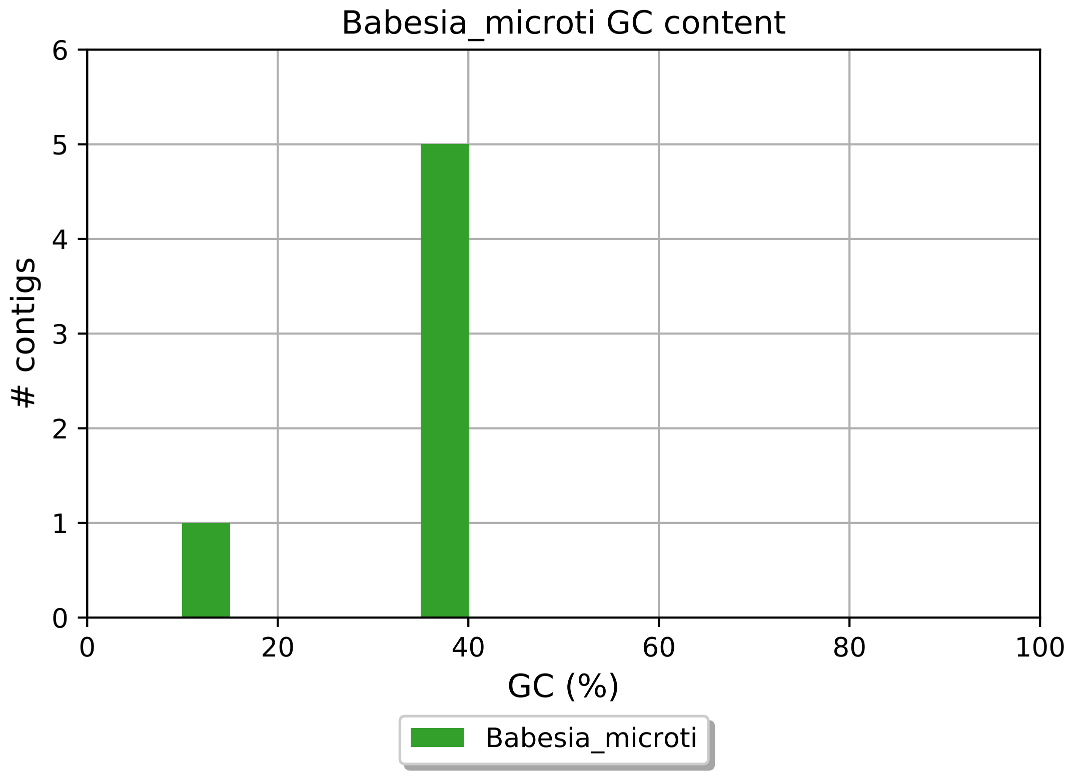
Babesia_microti GC content
contigs
GC (%)
0
1
2
3
4
5
6
0
20
40
60
80
100
Babesia_microti
