# Supplementary figures and images for: Babesia gibsoni Whole-Genome Sequencing, Assembling, Annotation, and Comparative Analysis
Source: Microbiol Spectr. 2023 Jul 11;11(4):e00721-23. doi: 10.1128/spectrum.00721-23 (PMC10434002; doi:10.1128/spectrum.00721-23)

Figure S2 KEGG pathway enrichment and GO annotation of nuclear genome

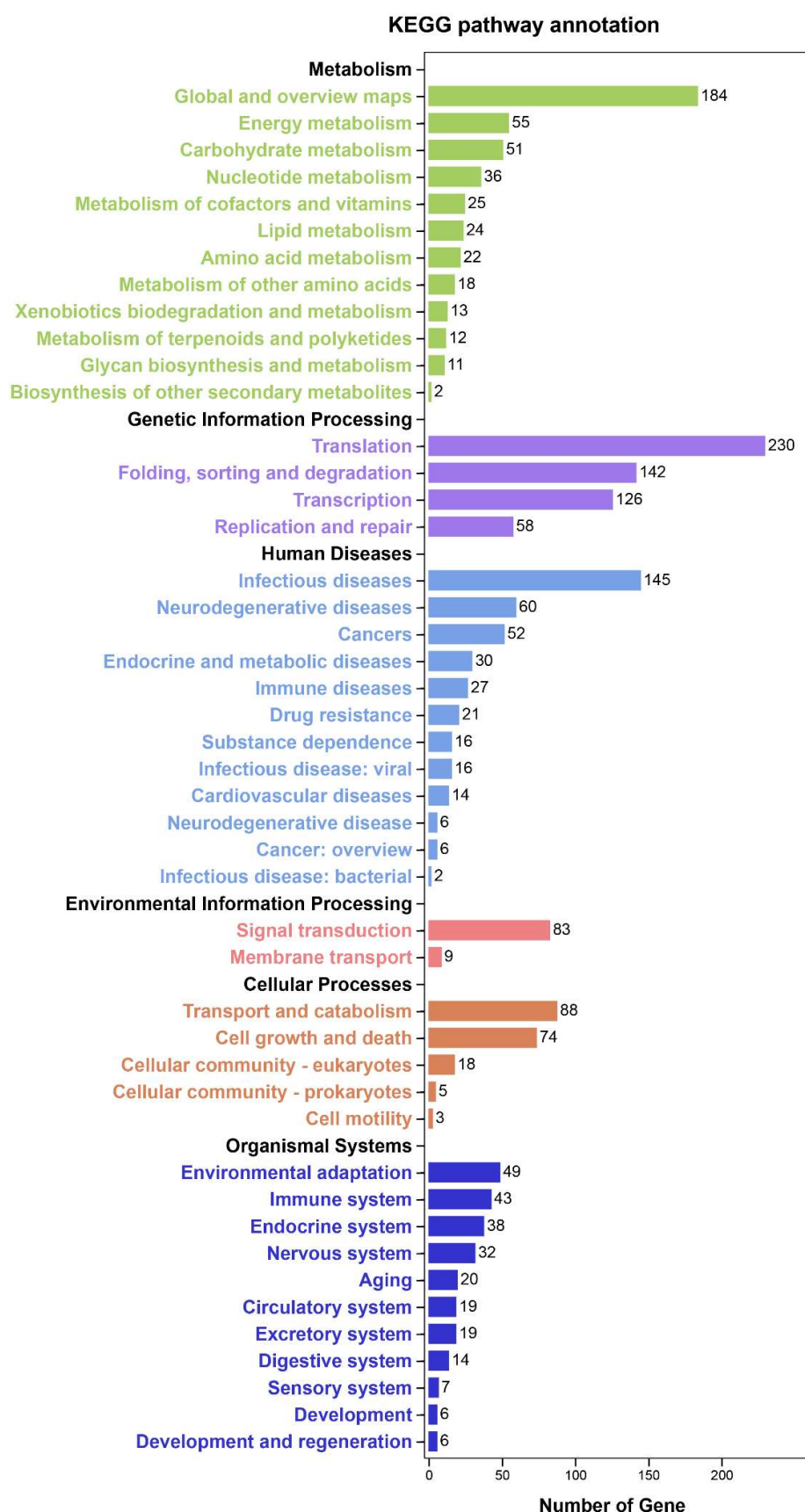

## GO annotation

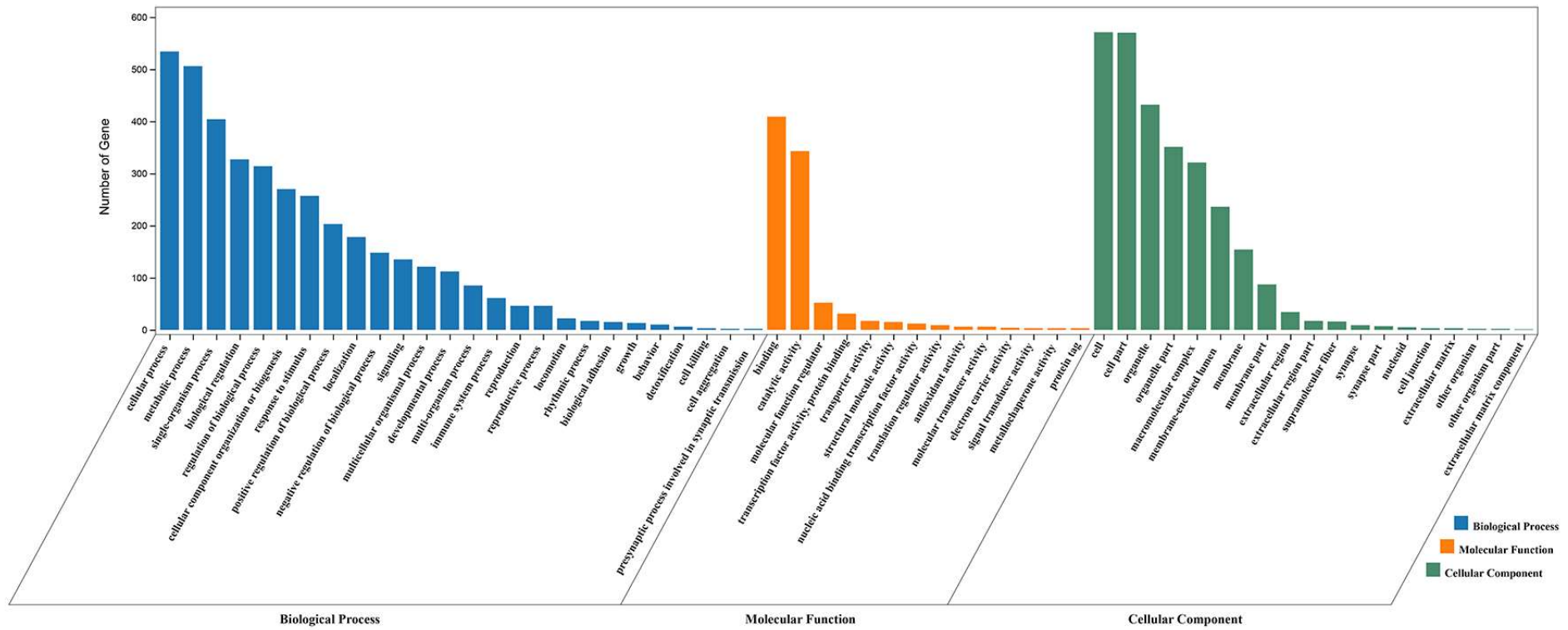

Supplement: Supplemental file 2 — Figure S2. Download spectrum.00721-23-s0002.pdf, PDF file, 0.5 MB [file spectrum.00721-23-s0002.pdf]
