# Supplementary material for: Babesia gibsoni Whole-Genome Sequencing, Assembling, Annotation, and Comparative Analysis
Source: Microbiol Spectr. 2023 Jul 11;11(4):e00721-23. doi: 10.1128/spectrum.00721-23 (PMC10434002; doi:10.1128/spectrum.00721-23)
Supplement: Supplemental file 3 — Table S1. Download spectrum.00721-23-s0003.docx, DOCX file, 0.01 MB [file spectrum.00721-23-s0003.docx]

Table S1 Quality test of *B. gibsoni* DNA samples

| Sample | Peak figure | Degradation | Contamination | OD260/280 | OD260/230 | Nanodrop/Qubit | Source |
| --- | --- | --- | --- | --- | --- | --- | --- |
| bg1 | Normal | Mild | - | 1.9 | 1.9 | 8.5 | Supernatant |
| bg2 | Normal | - | - | 1.92 | 1.87 | 5.1 | Sediment |
