# Supplementary material for: Babesia gibsoni Whole-Genome Sequencing, Assembling, Annotation, and Comparative Analysis
Source: Microbiol Spectr. 2023 Jul 11;11(4):e00721-23. doi: 10.1128/spectrum.00721-23 (PMC10434002; doi:10.1128/spectrum.00721-23)
Supplement: Supplemental file 4 — Table S2. Download spectrum.00721-23-s0004.docx, DOCX file, 0.02 MB [file spectrum.00721-23-s0004.docx]

Table S2 RNA-seq information of four chromosomes within *B. gibsoni*.

| Attribute | Chr | Start | End | score | +/- |
| --- | --- | --- | --- | --- | --- |
| 5s_rRNA | ChrIV | 1057130 | 1057247 | 75.2 | + |
|  | ChrIV | 1241793 | 1241910 | 75.2 | + |
|  | ChrIV | 1247958 | 1248075 | 75.2 | + |
|  | ChrIV | 1235953 | 1236070 | 75.2 | - |
| 18s_rRNA | ChrIV | 404914 | 406624 | 1426.5 | + |
|  | ChrII | 664804 | 666514 | 1426.5 | - |
| 28s_rRNA | ChrIV | 407035 | 414201 | 3044.7 | + |
|  | ChrII | 657277 | 664393 | 3043.1 | - |
