# Supplementary material for: Babesia gibsoni Whole-Genome Sequencing, Assembling, Annotation, and Comparative Analysis
Source: Microbiol Spectr. 2023 Jul 11;11(4):e00721-23. doi: 10.1128/spectrum.00721-23 (PMC10434002; doi:10.1128/spectrum.00721-23)
Supplement: Supplemental file 5 — Table S3. Download spectrum.00721-23-s0005.docx, DOCX file, 0.01 MB [file spectrum.00721-23-s0005.docx]

Table S3 The prediction results of the tRNA of *B. gibsoni.*

| Chromosome | tRNA number | tRNAs with introns |
| --- | --- | --- |
| ChrI | 8 | 1 |
| ChrII | 10 | 0 |
| ChrIII | 12 | 0 |
| ChrIV | 16 | 0 |
