# Supplementary material for: Babesia gibsoni Whole-Genome Sequencing, Assembling, Annotation, and Comparative Analysis
Source: Microbiol Spectr. 2023 Jul 11;11(4):e00721-23. doi: 10.1128/spectrum.00721-23 (PMC10434002; doi:10.1128/spectrum.00721-23)
Supplement: Supplemental file 8 — Table S6. Download spectrum.00721-23-s0008.docx, DOCX file, 0.02 MB [file spectrum.00721-23-s0008.docx]

Table S6 Information table of 20 hypothetical proteins annotated as secreted antigens

| No. | Gene ID | Query Cover | E value | Per. Ident | Annotation |
| --- | --- | --- | --- | --- | --- |
| 1 | BgWH_03g00237 | 100% | 0 | 100.00% | NR: secreted antigen 1, partial (*Babesia gibsoni*) |
| 2 | BgWH_03g00003 | 70% | 2.00E-170 | 56.78% | NR: secreted antigen 1 (Babesia gibsoni) |
| 3 | BgWH_03g00130 | 76% | 1.00E-161 | 51.84% | NR: secreted antigen 1 (*Babesia gibsoni*) |
| 4 | BgWH_03g00212 | 76% | 1.00E-161 | 51.84% | NR: secreted antigen 1 (*Babesia gibsoni*) |
| 5 | BgWH_03g00234 | 72% | 4.00E-155 | 49.42% | NR: secreted antigen 3 (*Babesia gibsoni*) |
| 6 | BgWH_03g00235 | 76% | 9.00E-141 | 48.74% | NR: secreted antigen 1 (*Babesia gibsoni*) |
| 7 | BgWH_03g00236 | 94% | 1.00E-134 | 40.32% | NR: secreted antigen 3 (*Babesia gibsoni*) |
| 8 | BgWH_03g00238 | 75% | 3.00E-130 | 43.36% | NR: secreted antigen 3 (*Babesia gibsoni*) |
| 9 | BgWH_03g00809 | 93% | 2.00E-127 | 39.47% | NR: secreted antigen 3 (*Babesia gibsoni*) |
| 10 | BgWH_03g01228 | 100% | 2.00E-112 | 33.82% | NR: secreted antigen 1, partial (*Babesia gibsoni*) |
| 11 | BgWH_03g01229 | 63% | 1.00E-59 | 33.51% | NR: secreted antigen 1 (*Babesia gibsoni*) |
| 12 | BgWH_02g00158 | 73% | 8.00E-35 | 24.95% | NR: secreted antigen 1, partial (*Babesia gibsoni*) |
| 13 | BgWH_02g00159 | 59% | 2.00E-30 | 26.46% | NR: secreted antigen 3 (*Babesia gibsoni*) |
| 14 | BgWH_02g00160 | 61% | 2.00E-22 | 23.08% | NR: secreted antigen 1, partial (*Babesia canis canis*) |
| 15 | BgWH_02g00161 | 57% | 1.00E-19 | 23.80% | NR: secreted antigen 1, partial (*Babesia gibsoni*) |
| 16 | BgWH_01g00258 | 60% | 5.00E-19 | 24.42% | NR: secreted antigen 1, partial (*Babesia canis canis*) |
| 17 | BgWH_04g00039 | 53% | 4.00E-16 | 22.66% | NR: secreted antigen 3 (*Babesia gibsoni*) |
| 18 | BgWH_04g00040 | 58% | 7.00E-16 | 21.83% | NR: secreted antigen 1, partial (*Babesia canis canis*) |
| 19 | BgWH_04g00510 | 58% | 1.00E-12 | 21.87% | NR: secreted antigen 1, partial (*Babesia canis canis*) |
| 20 | BgWH_04g00735 | 50% | 7.00E-11 | 22.12% | NR: secreted antigen 1, partial (*Babesia canis canis*) |
